# Supplementary material for: The fishery performance indicators for global tuna fisheries
Source: Nat Commun. 2019 Apr 9;10:1641. doi: 10.1038/s41467-019-09466-6 (PMC6456575; doi:10.1038/s41467-019-09466-6)
Supplement: Supplementary file 1 — Supplementary Information [file 41467_2019_9466_MOESM1_ESM.pdf]

Supplementary Information for

The Fishery Performance Indicators for Global Tuna Fisheries

McCluney et al.

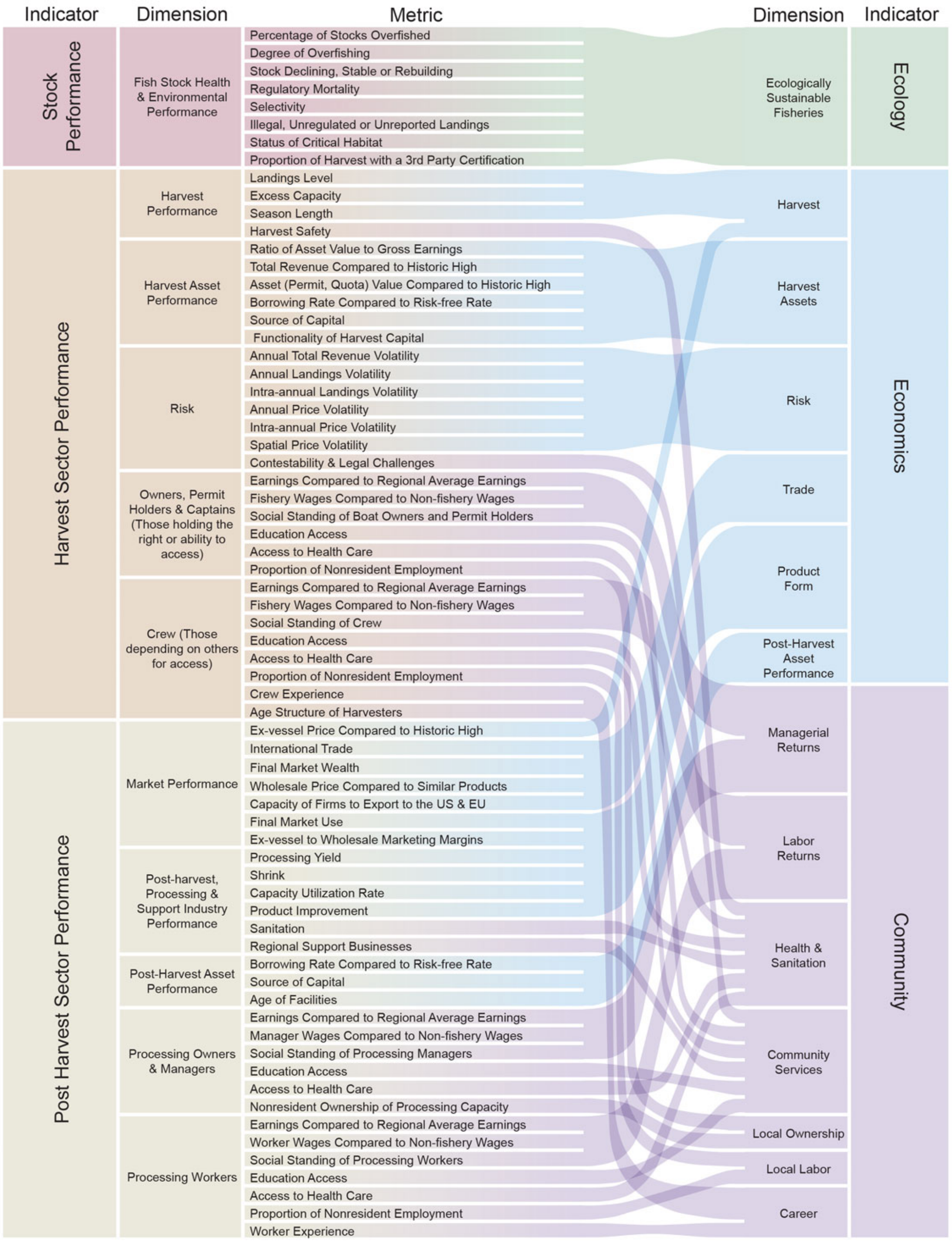

Supplementary Figure 1 Schematic of the Fishery Performance Indicators outcome measures, dimensions and indicators for the Stock Performance, Harvest Sector Performance, and Post-Harvest Sector Performance indicators.

Component	Dimension	Measure
Macro Factors	General Environmental Performance	Environmental Performance Index (EPI)
	Exogenous Environmental Factors	Disease and Pathogens
		Natural Disasters and Catastrophes
		Pollution Shocks and Accidents
		Level of Chronic Pollution (Stock effects)
		Level of Chronic Pollution (Consumption effects)
	Governance	Governance Quality
		Governance Responsiveness
	Economic Conditions	Index of Economic Freedom
		Gross Domestic Product (GDP) Per Capita
Property Rights & Responsibility	Fishing Access Rights	Proportion of Harvest Managed Under Limited Access
		Transferability
		Security
		Durability
		Flexibility
		Exclusivity
	Harvest Rights	Proportion of Harvest Managed with Rights-based Management
		Transferability
		Security
		Durability
		Flexibility
		Exclusivity
Co-Management	Collective Action	Proportion of Harvesters in Industry Organizations
		Harvester Organization Influence on Fishery Management & Access
		Harvester Organization Influence on Business & Marketing
	Participation	Days in Stakeholder Meetings
		Industry Financial Support for Management
	Community	Leadership
		Social Cohesion
	Gender	Business Management Influence
		Resource Management Influence
		Labor Participation in Harvest Sector
		Labor Participation in Post-Harvest Sector
Management	Management Inputs	Management Expenditure to Value of Harvest
		Enforcement Capability
		Management Jurisdiction
		Level of Subsidies
	Data	Data Availability
		Data Analysis
	Management Methods	MPAs and Sanctuaries
		Spatial Management
		Fishing Mortality Limits
Post-Harvest	Markets & Market Institutions	Landings Pricing System
		Availability of Ex-vessel Price & Quantity Information
		Number of Buyers
		Degree of Vertical Integration
		Level of Tariffs
		Level of Non-tariff Barriers
	Infrastructure	International Shipping Service
		Road Quality
		Technology Adoption
		Extension Service
		Reliability of Utilities/Electricity
		Access to Ice & Refrigeration

Supplementary Figure 2 Schematic of Fishery Performance Indicator enabling conditions measures, dimensions and components.

Supplementary Table 1 Detailed Descriptions of tuna fisheries by t-RFMO regions. Summary of key information describing each fishery scored. Columns are presented in the same order as shown in Table 1. The first row contains information about exactly what was scored: the percentage of the total catch of the fishery is represented by the directly scored fleet; the authors' subjective confidence in the scores in the case study and their representativeness of the overall fleet; and the specific fleets that were scored. Since the RFMOs manage biological stocks, it can often be difficult to divide catch of a species among fleets, especially small scale fleets on which there is not precisely tracked data, and some tonnages and catch composition information is divided among fleets that target the same stocks based on available information. Where possible, proportions are based on the 2012 ISSF Stock Rankings Report giving catch by RFMO and gear type. RFMO databases and FAO reports were utilized when ISSF did not have information on a particular FPI fishery. Data reports in these studies ranged from 2009-2012, so weighting quantities may not be drawn from the same year.

Western and Central Pacific Fisheries Commission – Purse Seine Fisheries

**Fishery**	**WCPO Distant Water Purse Seine—Asian Fleet (I DWPS)**	**WCPO Industrial Purse Seine– Local non-PNA Fleet (I PS)**	**WCPO Industrial Purse Seine – FSMA Fleet (I PS)**	**WCPO Distant Water Purse Seine – US Fleet (I DWPS)**
Proportion of regional catch on which scoring is based; A-C confidence	~65% of **935,000t**; B<br>Based on Japanese and Taiwanese fleets in various PIC transshipment ports and home ports	~40% of **551,000t**; C<br>Based on Indonesia landing in East Java, Jakarta Indonesia.	>75% of **361,000t**; B<br>Based on PNG, Marshall Islands and Solomon Islands fleets in Madang, Majuro and Honiara..	100% of **282,000t**; A<br>Based on 'old' and 'new' US fleets in Pago Pago, American Samoa, and various PIC transshipment ports
Catch composition	Tuna-targeted fishery (~1/2 the PS catch is made by fishing on FADs in this region); In general, purse seiners catch:<br>~86% of the WCPO-SKJ stock<br>~71% of the WCPO-YFT stock<br>~38% of the WCPO-BET stock (many juveniles)	Tuna-targeted fishery (~1/2 the PS catch is made by fishing on FADs in this region); In general, purse seiners catch:<br>~86% of the WCPO-SKJ stock<br>~71% of the WCPO-YFT stock<br>~38% of the WCPO-BET stock (many juveniles)	Tuna-targeted fishery (~1/2 the PS catch is made by fishing on FADs in this region); In general, purse seiners catch:<br>~86% of the WCPO-SKJ stock<br>~71% of the WCPO-YFT stock<br>~38% of the WCPO-BET stock (many juveniles)	Tuna-targeted fishery (~1/2 the PS catch is made by fishing on FADs in this region); In general, purse seiners catch:<br>~86% of the WCPO-SKJ stock<br>~71% of the WCPO-YFT stock<br>~38% of the WCPO-BET stock (many juveniles)
Significant bycatch[1]	Moderate incidence of incidental mammal, shark, sea bird, and sea turtle catch associated with FAD-fishing.	Moderate incidence of incidental mammal, shark, sea bird, and sea turtle catch associated with FAD-fishing.	Moderate incidence of incidental mammal, shark, sea bird, and sea turtle catch associated with FAD-fishing.	Moderate incidence of incidental mammal, shark, sea bird, and sea turtle catch associated with FAD-fishing.

[1] Many small-scale tuna fisheries opportunistically target tuna depending on the season. Because we sought to capture the socio-economic aspects of the fishery's performance in addition to the ecological status, in fisheries where tuna is prosecuted opportunistically, seasonally, or otherwise supplemented with fishing for other species (usually large pelagics), we considered tuna as a component of a multi-species fishery in which there was no true bycatch.

Season	Year-round with 3-month bans from fishing on FADs within PNA EEZs	Year-round with 3-month bans from fishing on FADs within PNA EEZs (difficult to apply to Indonesia and Philippines who mainly fish within their archipelagic waters)	Year-round with 3-month bans from fishing on FADs within PNA EEZs	Year-round with 3-month bans from fishing on FADs within PNA EEZs
Structure of management and compliance responsibility	VD residual from FSMA and US Treaty portions allocated based on competitive prices negotiated in bilateral agreements. Comply with WCPFC stock measures, flag-state mandated fishery regulations	Most catch in own EEZ, without limits on capacity. Comply with WCPFC stock measures, flag-state mandated fishery regulations	VD allocation priority for domestic fleets. Comply with WCPFC stock measures, flag-state mandated fishery regulations	VD allocation is determined by treaty, with prices (below those paid by the Asian fleet) by multilateral agreement. Comply with WCPFC stock measures, flag-state mandated fishery regulations
Type of vessels	~1400-2000GRT, steel, decked, RSW, inboard diesel engines; brand new in Taiwan	<<1000GRT, reinforced fiberglass, wood, steel, decked, RSW, freezer capacity, inboard diesel engines; usually gifted	~1000GRT, steel, decked, RSW, inboard diesel engines; usually foreign capital or gifted	~1400-2000GRT, steel, decked, RSW, inboard diesel engines; brand new in Taiwan
Approximate fleet size	59 Ecuador flag and 12 Panama flag vessel class size 4-6	345 registered in Jakarta alone	<50	37; 18 'old', 19 'new' (up to 40 under US Treaty)
Approximate number of officers, crew aboard vessels	25-35 including officers (officers are Taiwanese nationals but crew is usually mostly Indonesian, Filipino, SE Asian)	35-40 including officers (usually flag state nationals)	12-25 including officers (officers might be Pacific Islander national or Asian but crew is usually mostly Indonesian, Filipino, SE Asian)	25-35 including officers (officers are US or Taiwanese, Korean nationals but crew is usually mostly Indonesian, Filipino, SE Asian)
Primary markets	Global canned market (US, EU, Middle East, Oceana) In 2012, PS SKJ from free unassociated schools (no FADs) in PNA waters were MSC certified.	Large domestic markets (230 million to feed on Java alone)	Global canned market (US, EU, Middle East, Oceana)	Global canned market (US, EU, Middle East, Oceana)) In 2012, PS SKJ from free unassociated schools (no FADs) in PNA waters were MSC certified.
Processing plants	Thai canneries operating at combined 2500t/day (3000t capacity). Thai Union – largest at 1000t/day; Sea Value 850t; remaining companies are medium-scale firms (~1200t combined). 1000s of jobs.	3-6 smaller scale canneries on Java	Ranges from two plants between Majuro and Honiara processing 40-60t/day; operating >50% undercapacity and reported labor problems; large Thai canneries with 1000s of jobs; PNG canneries with Filipino capital and reportedly contentious labor conditions	1 cannery still in Pago Pago to qualify final product as duty-free, but operating under capacity with reported labor problems

Generalized steps in the supply chain	95% to Thai canneries through one of three tuna trading companies, use of 8 regional transshipment ports; Japanese fleet supplies domestic canneries	Much supplies Thai canneries through tuna trading companies but ~90,000t supply domestic canneries for local consumption	Landings supply PIC canneries. Some loining in Majuro and exported to Pago Pago, but overall, majority of metafishery landings are exported to Thai canneries; both Honiara and Majuro (and 6 others) are transshipment ports	Some still supply cannery in Pago Pago but most supplies Thai canneries through one of three tuna trading companies, use of 8 regional transshipment ports
Qualitative trends in landings	Increasing	Both Indonesia and Philippines have increased in the past 5 years	Landings for Solomon and Marshall Islands steadily decreasing while PNG exponentially rising over the past decade	Resurgence in the past decade

Western and Central Pacific Fisheries Commission – Longline Fisheries

**Fishery**	**WCPO Distant Water Longline (Frozen) (I DWLL)**	**WCPO Industrial Longline (Fresh) (I LL)**	**WCPO Artisanal Longline/Handline (A LL/HL)**	**WCPO Industrial Longline – Local Fleet (I LL)**
Proportion of regional catch on which scoring is based; A-C confidence	>75% of **151,000**; A<br>Based on Japanese and Taiwanese frozen sashimi fleets in various ports in Japan, and Kaohsiung, Taichung, Taiwan	>90% of **65,000t**; A<br>Based on Japanese and Taiwanese fresh sashimi fleets in various ports in Japan, and Kaohsiung, Taichung, Taiwan	<10% **of 38,000t** B<br>Based on two village-fleets landing in the Banda Sea region, Indonesia	~80% of **14,000t**; B<br>Based on the MSC-certified Fijian fleet in Suva
Catch composition	Tuna-targeted fishery; In general longliners catch:<br>~51% of the WCPO-BET stock<br>~12% of the WCPO-YFT stock<br>~95% of the S-PO-ALB stock<br>~40% of the N-PO-ALB stock<br>~12% of the PO-BFT stock<br>(~55% of the SBFT stock, see CCBST fisheries)	Tuna-targeted fishery; In general longliners catch:<br>~51% of the WCPO-BET stock<br>~12% of the WCPO-YFT stock<br>~95% of the S-PO-ALB stock<br>~40% of the N-PO-ALB stock<br>~12% of the PO-BFT stock<br>(~55% of the SBFT stock, see CCBST fisheries)	Seasonal tuna fishery;<br>In general longliner/handliner catch:<br>~51% of the WCPO-BET stock<br>~12% of the WCPO-YFT stock	Tuna-targeted fishery; In general longliners catch:<br>~95% of the S-PO-ALB stock (70% MSC-certified)<br>~12% of the WCPO-YFT stock<br>~51% of the WCPO-BET stock
Significant bycatch	Low incidence of incidental mammal, sea bird, and sea turtle catch associated with this gear type. Mitigation measures exist.	Low incidence of incidental mammal, sea bird, and sea turtle catch associated with this gear type. Mitigation measures exist.	Very low incidence of incidental mammal, shark, sea bird, and sea turtle catch associated with this gear type.	Low incidence of incidental mammal, sea bird, and sea turtle catch associated with this gear type. Mitigation measures exist.

Season	Year-round	Year-round	7 months; Nov-Dec peak season, March-May and Sept-Oct are buffer months	Year-round
Structure of management and compliance responsibility	Comply with WCPFC stock measures, flag-state mandated fishery regulations	Comply with WCPFC stock measures, flag-state mandated fishery regulations	Comply with WCPFC stock measures, flag-state mandated fishery regulations	Comply with WCPFC stock measures, flag-state mandated fishery regulations
Type of vessels	>100GRT, >24m LOA, steel, decked, RSW and ULT freezer, inboard diesel engine	Most ~80GRT,range 20-120GRT <24m LOA, steel, decked, RSW and ULT freezer, inboard diesel engine	8m LOA, reinforced fiberglass, wood, undecked, no cold storage capacity, outboard motors	~24m LOA, steel, decked, RSW, inboard diesel engines
Approximate fleet size	~600	1400-2000	100,000s	97 (in 2009)
Approximate number of officers, crew aboard vessels	18-25 including officers (officers are flag state nationals but crew is usually mostly Indonesian, Filipino, SE Asian)	12-20 including officers (officers are flag state nationals but crew is usually mostly Indonesian, Filipino, SE Asian)	1 captain, 0 or 1 or 2 crew (all flag state national)	5-15 including officers (only a fraction are local; crew is usually mostly Indonesian, Filipino, SE Asian)
Primary markets	Japanese sashimi	Japanese sashimi	Mainly US fresh/frozen (EU-ban on CO)	EU as MSC-frozen steaks; US, Japan sashimi; China, Australia fresh/frozen market
Processing plants	Minimal processing to prepare sashimi bullets – done aboard vessels; 700 trading companies and 5 auction houses in Tsukiji; some ALB will go to Thai canneries or Pago Pago	Minimal processing to prepare sashimi bullets –5 auction houses in Tsukiji handling 80% sashimi for Japan	Fresh/frozen export plants sprinkled throughout region; e.g. 1 Ambon, 2-3 Flores, etc.; on average ~50 jobs each	Three major domestic export companies, pretty well vertically integrated; fresh/frozen products; 100s of jobs
Generalized steps in the supply chain	Frozen onboard and transshipped at sea, as fishing vessels stay out 18-24 months; upon landing, 70-80% lower quality-BET,YFT, ALB is sold through trading companies and processors directly from vessel; 20-30% higher quality-BET and BFT passes through auction system. Not uncommon for trading companies to store product for 1-2 years. Distribution has become very complicated.	Chilled onboard. Lower instances of transshipping than frozen fleet; most fresh still goes through highly complex traditional auction system on behalf of vessels owners to then wholesalers although increasingly moving to direct sales to larger retailers	"Suppliers" collect tuna landed at beachside ceramic huts (*klostors*) and transport by truck to a local plant branch; then gets transported to an export plant destined for US market (usually)	Direct from Fiji to export market for disbursement
Qualitative trends in landings	Stagnant prices; 1/3 vessels are profitable, 1/3 are borderline, the rest are struggling and on the road to bankruptcy (industry source)	Taiwanese business model allows their fleet to be one of the most economically successful; offshore LL fleets on average barely break even	Recorded landings have jumped since 2010 because of implementation of a recent reporting initiative	Landings stable but prices severely undercut by Chinese LL; predicted financial collapse in 2013

Indian Ocean Tuna Commission

**Fishery**	**IO Semi-industrial Gillnet/ Longline (SE GN/LL)**	**IO Distant Water Purse Seine (I DWPS)**	**IO Semi-industrial Longline/Handline (SE LL/HL)**	**IO Semi-industrial Pole-and-line (MSC Certfiied) (SE PL)**
Proportion of regional catch on which scoring is based; A-C confidence	<15% of **303,480t**; C<br>Based on Sri Lankan fleet in Negombo, Sri Lanka	>90% of **243,000**; A<br>Based on EU fleet in Port Victoria, Seychelles	~30% of **119,000t**; B<br>Based on Sri Lankan, Maldivian, IO-Indonesian, Seychellois fleets landing locally	>90% of **110,000t**; A<br>Based on Maldivian fleet in Laamu Gan, Gaafu Alif (southern atolls),Maldives
Catch composition	Multiple neritic species targeted;<br><15% IO-SKJ<br><10% IO-YFT (juvenile)	Tuna-targeted fishery (Spanish set heavily on FADs, French set heavily on free schools – slightly more variance in SKJ:YFT proportions due to these activities);<br>60-80% IO-SKJ<br>20-40% IO-YFT<br>some IO-BET (many juvenile)	Often multiple pelagic species targeted; tuna 30-100% catch depending on season, fleet<br>70-80% IO-YFT<br>20-30% IO-BET	Tuna-targeted fishery;<br>90% IO-SKJ<br>10% IO-YFT (juvenile)
Significant bycatch	High incidence of sharks, mammals, sea turtles associated with gear method	Low incidence of incidental mammal, seabird, shark, sea turtle catch associated with this gear type	Very low incidence of incidental mammal, sea turtle catch associated with this gear type. Sea bird mitigation measures exist at RFMO level.	Scad, reef fish targeted for bait; very low incidence of incidental mammal, seabird, shark, sea turtle catch associated with this gear type
Season	Year-round	Year-round	Tuna seasonal; fishing occurs year-round	Year-round
Structure of management and compliance responsibility	Comply with IOTC stock measures, flag-state mandated fishery regulations	Comply with IOTC stock measures, flag-state mandated fishery regulations	Comply with IOTC stock measures, flag-state mandated fishery regulations	Comply with IOTC stock measures, flag-state mandated fishery regulations
Type of vessels	~30GT, 10-15m LOA, wood/fiberglass, decked with an ice hold, inboard diesel engine	1000-1400 GRT, ~100m LOA, reinforced steel, multiple deck levels, RSW, inboard twin diesel engines	~30GT, <24m LOA, wood/fiberglass, decked with an ice hold, inboard diesel engine	20-25m LOA, wood/fiberglass, decked with an ice hold, inboard diesel engine
Approximate fleet size	10,000s (3000 in Sri Lanka alone)	~32 Spanish vessels and ~17 French vessels	1000s (~800 between Maldives and Sri Lanka alone)	500-1000
Approximate number of officers, crew aboard vessels	1 captain, 4-5 crew (all flag state nationals)	9 officers (EU), 24 crew (African)	1 captain, 5-15 crew (all flag state nationals)	1 captain, 12-35 crew (all Maldivian)
Primary markets	Local	EU and UK canned export	US, EU, Japan fresh/frozen export	UK, EU canned export
Processing plants	N/A	IOT, Ltd employs 2400 in Seychelles, 350t throughput, 1.5 million cans produced. Another smaller cannery in Mauritius.	2-14 depending on the country, employs 20-120, processing ranges from minimal for fresh chilled export to frozen filleting for saku, steaks for export	4 main companies; ~1000 employees, largest is government-owned

Generalized steps in the supply chain	Sold whole round upon landing at local markets	EU fleet offloads to Seychelles for primary processing through canned/ pouched product for export.	Primary processing occurs upon landing in flag state country, participating directly in international market	10% is landed by a private company that cans the product and utilizes the MSC-certified skipjack label for the UK market. 90% of raw product is landed and exported to Thailand for primary processing.
Qualitative trends in landings	% tuna varies seasonally in this opportunistic fishery	Increasing since 2012; piracy had driven out fleet from 2010-2012	% tuna varies seasonally in this opportunistic fishery	Decreasing since 2007

Inter-American Tropical Tuna Commission

**Fishery**	**EPO Industrial Purse Seine (I PS)**	**EPO Industrial Pole-and-Line/Troll (MSC Certified) (I PL/TR)**	**EPO Industrial Bluefin Purse Seine/Ranching (I PS/RN)**	**EPO Artisanal Longline (A LL)**
Proportion of regional catch on which scoring is based; A-C confidence	~50% of **454,020t**; B<br>Based on Ecuadorian/Panamanian fleets in Manta, Ecuador	>80% of **40,869t**; A<br>Based on US fleet in Washington, Oregon, USA	~80% of **10,000t**; A<br>Based on Mexican fleets and ranching operations in Ensenada, Mazatlan, Mexico	>80% of **11,000t**; A<br>Based on Ecuadorian *fibras*[2] fleets in Manta, Santa Marianita, San Mateo, Ecuador
Catch composition	Tuna-targeted fishery, about 70:30 SKJ:YFT; In general purse seiners catch:<br>99% of the EPO-SKJ stock (34% free schools, 64% FADs)<br>~97% of the EPO-YFT stock (17% free schools, 17% FADs, 62% dolphin-associated schools)<br>~70% of the EPO-BET stock (virtually all while on FADs)	Tuna-targeted fishery; In general pole-and-liners catch 33% of the N-PO-ALB stock.	Purse seiners catch ~64% of the PO-BFT stock<br>Varying seasons of high juvenile catch	Known as the Tuna-Billfish-Shark fishery; tuna only makes up ~10% of this fishery; In general, longliners catch:<br>~3% of the EPO-YFT stock<br>~29% of the EPO-BET stock
Significant bycatch	Very low incidence of incidental seabird, shark, sea turtle catch associated with this gear type. Virtually zero incidence of dolphin mortality. 100% observer coverage.	Baitfish; very low incidence of incidental mammal, seabird, shark, sea turtle catch associated with this gear type	Feed fish (small pelagics); Very low incidence of incidental mammal, shark, sea bird, and sea turtle catch associated with this gear type.	Low incidence of mammals, sea birds associated with gear method. Regional mitigation measures for sea turtles exist.

[2] *Fibras* participating in the tuna-billfish-shark fishery are usually associated with a *nadrisa*, or mothership. For the scope of this pilot project, the *nadrisas* were not directly scored, yet their influence on *fibras* performance is indirectly incorporated.

Season	Year-round minus a 2 month closure	Year-round	Usually 5-6 weeks	May – October
Structure of management and compliance responsibility	Comply with IATTC stock measures, flag-state mandated fishery regulations	Comply with IATTC stock measures, flag-state mandated fishery regulations	Comply with IATTC stock measures, flag-state mandated fishery regulations	Comply with IATTC stock measures, flag-state mandated fishery regulations.
Type of vessels	>363-~2800GRT; average 1200-1600GRT, reinforced steel, multiple deck levels, RSW, inboard twin diesel engines, speedboats and helicopters on many vessels	20-25m LOA, steel, decked with RSW or freezer capacity, inboard diesel engine	Industrial purse seiners (~1000GRT, steel, decked, inboard diesel engines) with netting specialized for corralling tuna unharmed into ranching pens	6-8m LOA, wood/fiberglass, undecked, no cold storage capacity, 75HP outboard motors; generally towed to and from fishing grounds
Approximate fleet size	59 Ecuador flag and 12 Panama flag vessel class size 4-6	<200	<5 (one contracting company in Mexico)	10,000s (~15,000 in Ecuador alone)
Approximate number of officers, crew aboard vessels	4-6 officers, 18-20 crew (all mostly Latin American nationals)	2-4 officers, 8-12 crew (all American nationals)	6-10 officers and dive team, 8-14 crew (all Mexican fleet are nationals)	1 captain, 1-2 crew (local national)
Primary markets	EU and Latin American canned export	EU loined export, US domestic	95% Japanese sashimi	US, EU, Japanese fresh/frozen export; rejected tuna goes to the local markets
Processing plants	Several large canneries in Manta and Guayaquil; ~12,000 jobs	Most primary processing done in small plants in Washington, Oregon	Three ranching operations[3] in Mexico; minimal processing for chilled export; 100s of seasonal jobs, excellent pay by local standards	Several fresh/frozen export plants in Manta processing tuna and large pelagics; local vendors sell export rejects at market; 1000s of jobs
Generalized steps in the supply chain	Primary processing 80% cans and loins for Latin American, EU market, 20% loined and exported to EU for canning; minor amount to US	Majority of landings are loined and exported to Spain for secondary processing who does not pay the MSC premium; small boutique US market for fresh steaks	Tuna spend 10 months in fattening pens being held until best market price; exported fresh chilled to Japan	High quality catch gets processed into fresh/frozen products for export; poor quality catch gets steaked, filleted at local markets
Qualitative trends in landings	Total purse seine landings slightly increasing	No extraordinary changes recently	2012 saw the highest number of recruits since the 1980s, according to industry sources	% tuna varies seasonally in this opportunistic fishery

International Commission for the Conservation of Atlantic Tunas

**Fishery**	**AO Distant Water Purse Seine (I PS)**	**AO Distant Water Longline (I DWLL)**	**Mediterranean Industrial Bluefin Purse Seine/Trap Ranching (I PS/TP)**

[3] "Processing" for this fishery was reinterpreted to apply to the farming process of fattening tuna before selling on the market.

Proportion of regional catch on which scoring is based; A-C confidence	>60% of **198,000t**; B<br>Based on EU fleet in conjunction with the Ghanaian baitboats in Tema, Ghana	>60% of **57,414t** of frozen and >80% of **24,606t** of fresh; B<br>Based on Japanese and Taiwanese frozen sashimi fleets in various ports in Japan, and Kaohsiung, Taichung, Taiwan	~80% of **6,780t**; B<br>Based on fleets and ranching operations in Turkey, Spain, Croatia, Malta, Greece, & Italy
Catch composition	Tuna-targeted fishery (Spanish set heavily on FADs, French set heavily on free schools);<br>~63% E-AO-SKJ<br>~7% W-AO-SKJ<br>~23% AO-YFT<br>~7% AO-BET (many juvenile)	Tuna-targeted fishery (% by landings);<br>~ 25% AO-YFT<br>~50% AO-BET<br>~4%N-AO-ALB<br>~13% S-AO-ALB<br><2% E/Med-AO-BFT<br>(<1% SBFT)	~60% of the E/Med-AO-BFT stock is caught with purse seine.<br>Varying seasons of high juvenile catch.
Significant bycatch	Low incidence of incidental mammal, seabird, shark, sea turtle catch associated with this gear type	Low incidence of incidental mammal, sea bird, and sea turtle catch associated with this gear type. Mitigation measures exist.	Feed fish (small pelagics); Very low incidence of incidental mammal, shark, sea bird, and sea turtle catch associated with this gear type.
Season	Year-round	Year-round	4 weeks (11-month closure)
Structure of management and compliance responsibility	Comply with ICCAT stock measures, flag-state mandated fishery regulations	Comply with ICCAT stock measures, flag-state mandated fishery regulations	Comply with ICCAT stock measures, flag-state mandated fishery regulations
Type of vessels	100-2000 GRT, ~20-80m LOA, reinforced steel, multiple deck levels, RSW, inboard twin diesel engines	Frozen fleet vessels >100GRT, >24m LOA; Fresh fleet Most ~80GRT,range 20-120GRT <24m LOA, steel, decked, RSW and ULT freezer, inboard diesel engine	Industrial purse seiners (~1000GRT, steel, decked, inboard diesel engines) with netting specialized for corralling tuna unharmed into ranching pens
Approximate fleet size	~100; 93 BET/YFT-target Spanish vessels and 10 French vessels registered	~600 in frozen fleet (~365 registered >20m), as many as 1400-2000 in fresh fleet, and many WCPO vessels fish here as well.	~110-180 vessels registered<br>54 ranching sites registered
Approximate number of officers, crew aboard vessels	9 officers (EU), 24 crew (African)	18-25 in larger frozen vessels; 12-20 in smaller fresh vessels including officers (officers are flag state nationals but crew is usually mostly Indonesian, Filipino, SE Asian)	6-10 officers and dive team, 8-14 crew

Primary markets	EU and UK canned export	Japanese sashimi	>95% Japanese sashimi
Processing plants	Pioneer Food Cannery employs 1300 in Tema, 20t loins, 800,000 cans produced.	Minimal processing to prepare sashimi bullets – done aboard vessels; 700 trading companies and 5 auction houses in Tsukiji; some ALB will go to Thai canneries	Several dozens of ranching operations[4] around Mediterranean; minimal processing for export; 1000s of seasonal jobs; harvest output usually ~1000-5000mt; feed conversion industry standard ~13:1 (sardines, small pelagics)
Generalized steps in the supply chain	EU fleet offloads to Tema or Abijdan, Ivory Coast for primary processing through canned/ pouched product for export.	Frozen is transshipped at sea, as fishing vessels stay out 18-24 months; upon landing, 70-80% lower quality-BET,YFT, ALB is sold through trading companies and processors directly from vessel; 20-30% higher quality-BET and BFT passes through auction system. Not uncommon for trading companies to store product for 1-2 years. Distribution has become very complicated.<br><br>Fresh fleet has lower instances of transshipping than frozen fleet; most fresh still goes through traditional auction system on behalf of vessels owners to then wholesalers although increasingly moving to direct sales to larger retailers	Tuna spend up to 3 years in fattening pens; exported fresh chilled or frozen to Japan
Qualitative trends in landings	Piracy in the Indian Ocean had caused more AO fishing from 2010-2012. Pressure has since been relieved as fleets return to the IO; now even cases of East African piracy threatening AO activity.	Individual quotas recently reduced due to discovery of severe underreporting past 10-20 years. Recent findings suggest stocks are recovering despite overfished status.	Individual quotas recently reduced due to discovery of severe underreporting past 10-20 years.

Commission for the Conservation of Southern Bluefin Tuna

**Fishery**	**SBFT Distant Water Longline (I DWLL)**	**Australian Industrial Bluefin Purse Seine/Ranching (I PS/RN)**
Proportion of regional catch on which scoring is based; A-C	>60% of **5,225t** ; A<br>Based on (mostly) Japanese and Taiwanese fresh and (mostly) frozen sashimi fleets in IO, AO, and PO. Japan holds	>95% of **4,275t**; A<br>Based on fleets and ranching operations in southern Australia.

[4] "Processing" for this fishery was interpreted to apply to the farming process of fattening tuna before selling on the market.

confidence	47% non-farming quota; Taiwan holds another 15%	
Catch composition	55-60% of the SBFT stock is caught by longline.<br><br>From longline fleets that target YFT, BET, ALB, and BFT in the IO, AO, and PO, some have quota to catch SBFT. ~80% of the SBFT is caught in the IO, then AO, followed by the WCPO (virtually none in the EPO). .	40-45% of the SBFT stock is caught by purse seine.<br><br>Varying seasons of high juvenile catch.
Significant bycatch	Low incidence of incidental mammal, sea bird, and sea turtle catch associated with this gear type. Mitigation measures exist.	Feed fish (small pelagics); Very low incidence of incidental mammal, shark, sea bird, and sea turtle catch associated with this gear type.
Season	Year-round	3-4 months (Jan-April)
Structure of management and compliance responsibility	Comply with CCSBT stock measures, flag-state mandated fishery regulations	Comply with CCSBT stock measures, flag-state mandated fishery regulations
Type of vessels	~80GRT or >100GRT, >20-24m LOA, steel, decked, RSW and ULT freezer, inboard diesel engine	Industrial purse seiners (~1000GRT, steel, decked, inboard diesel engines) with netting specialized for corralling tuna unharmed into ranching pens
Approximate fleet size	Japan 65 and Taiwan 33 registered SBFT vessels. Total including is 263 fishing SBFT.	16 AU vessels registered<br><br>49 ranching sites registered
Approximate number of officers, crew aboard vessels	12-25 (?) including officers (officers are flag state nationals but crew is usually mostly Indonesian, Filipino, SE Asian)	6-10 officers and dive team, 8-14 crew
Primary markets	Japanese sashimi	>90% Japanese sashimi, some domestic AU market
Processing plants	Minimal processing to prepare sashimi bullets – done aboard vessels; 700 trading companies and 5 auction houses in Tsukiji; handling 80% sashimi for Japan	13 ranching farm companies that fatten tuna after capture ; minimal processing for chilled export; 1000s of seasonal jobs; harvest output usually ~1000-5000mt; feed conversion industry standard ~13:1 (sardines, small pelagics)
Generalized steps in the supply chain	Transshipped at sea, as fishing vessels stay out 18-24 months; higher quality passes through highly complex traditional auction system on behalf of vessels owners to then wholesalers although increasingly moving to direct sales to larger retailers. Lower quality could end up in a trading company system. Not uncommon for trading companies to store product for 1-2 years. Distribution has become very complicated.	Tuna spend several months in fattening pens being held until harvest in mid-late winter; exported fresh chilled or frozen to Japan
Qualitative trends in landings	TAC has been rising since 2009.	TAC has been rising since 2009.

Supplementary Table 2 List of non-tuna FPI case studies used for comparison in Figure 1, grouped by industrial scale. Note that some fisheries are grouped with a different industrial scale than their title implies, because they self-identify as a scale that differs with the definition used in this paper.

Fishery	Country	Stock Performance	Harvest Sector Performance	Post-Harvest Sector Performance
**Artisanal**				
Pabna Sadullaspracole	Bangladesh	3.13	3.11	2.67
Beel Chatra	Bangladesh	3.50	4.18	2.67
Kailin Nadi	Bangladesh	3.38	3.39	2.67
Shrimp Artisanal	Colombia	2.25	3.16	3.68
Artisanal Sole and Catfish	Gambia	3.50	3.36	3.44
TRY Oysters	Gambia	3.00	2.23	1.78
Artisanal	Ghana	2.38	2.98	2.77
Artisanal Axim	Ghana	2.50	3.42	3.25
Lesser Sunda Artisanal Snapper	Indonesia	3.13	3.02	3.52
Indonesia Blue Crab	Indonesia	2.50	3.14	3.79
Suruga Pink Shrimp	Japan	4.38	3.90	3.99
Tokyo Bay	Japan	3.25	3.55	4.43
Artisanal Shimoni	Kenya	2.63	2.79	2.72
Octopus	Kenya	2.38	3.17	3.94
Artisanal Westpoint	Liberia	2.38	2.76	2.71
Artisanal Robertsport	Liberia	3.50	3.33	3.26
Lake Chiuta	Malawi	3.00	3.45	2.45
La Paz Bivalves	Mexico	2.38	2.56	2.94
La Paz Bivalves	Mexico	2.88	2.75	2.87
La Paz Bay Chocolata	Mexico	3.00	2.98	3.65
Philippines Blue Crab	Philippines	2.63	3.27	3.26
Artisanal Senegal	Senegal	2.50	3.16	3.76
Artisanal Ngaparou	Senegal	2.63	2.89	3.11
Inshore Artisanal	Seychelles	3.63	3.89	3.86
Sea Cucumber	Seychelles	3.00	3.56	4.19
Sherbro	Sierra Leone	2.63	3.33	2.66
Tombo	Sierra Leone	2.50	3.28	2.93
Lake Victoria Dagaa	Uganda	4.25	3.37	2.70
Lake Victoria Tilapia	Uganda	4.25	3.07	3.12
Nile Perch	Uganda	2.75	3.07	3.74

FL Spiny Lobster	US	3.75	3.65	3.97
CA Urchin10	US	3.50	3.61	3.92
Thanh Hoa	Vietnam	2.13	2.92	3.13
Southern Zone Rock Lobster	Australia	4.00	4.20	4.38
Western Zone Abalone	Australia	4.13	4.33	4.28
CA Nearshore Finfish	US	2.88	3.77	3.96
CA Sea Urchin	US	3.75	3.77	3.90
CA Sea Cucumber	US	3.50	3.58	3.78
Artisanal Westpoint2	Liberia	4.00	3.86	3.10
S. Sumatra blue swimming crab	Indonesia	3.13	3.10	4.00
SE Sulawesi blue swimming crab	Indonesia	2.63	2.95	4.00
Central Sulawesi demersal	Indonesia	2.50	3.45	4.04
S. Sulawesi grouper	Indonesia	2.50	3.18	3.39
N. Sumbawa demersal	Indonesia	2.75	2.98	3.36
S. Sumbawa demersal	Indonesia	2.38	2.68	3.32
**Semi-industrial**				
Shrimp Industrial	Colombia	2.38	3.19	4.00
Lobster (Nephrops)	Iceland	5.00	4.41	4.17
Semi-Industrial Liberia	Liberia	3.50	3.24	3.03
Central Zone	Morocco	3.06	3.78	3.41
Semi-Industrial Seychelles	Seychelles	3.50	4.16	3.98
Baltic Cod	Sweden	4.00	3.67	4.16
Louisiana Shrimp	US	3.50	3.52	3.75
AK Salmon	US	4.88	3.08	3.38
OR Dungeness Crab	US	4.63	3.38	3.50
AK Halibut	US	4.88	4.05	3.76
Pacific Groundfish	US	3.75	3.61	3.72
GOM Snapper	US	2.88	3.79	3.92
Fish Trawl (offshore)	Vietnam	2.25	3.33	3.49
Shrimp Trawl (inshore)	Vietnam	2.00	3.07	3.40
CA Spiny Lobster	US	3.25	3.64	3.92
CA Dungeness	US	4.13	3.58	3.95
CA Salmon	US	2.88	3.10	3.88
CA Market Squid	US	4.13	3.83	4.02
CA Spot Prawn	US	4.38	4.08	3.94
CA Rock Crab	US	3.75	3.74	3.88
CA Sablefish	US	4.00	3.62	3.88

Lombok shark	Indonesia	3.88	3.48	3.26
S. India Trawl	India	3.00	3.26	3.74
Gujarat Trawl	India	3.13	3.26	3.75
**Industrial**				
Spencer Gulf Prawn	Australia	4.38	4.09	4.17
Southern Zone	Morocco	3.69	4.14	3.38
Hoki	New Zealand	4.38	4.12	4.22
Cod	Norway	4.75	4.18	3.78
Purse Seiners	Norway	4.75	4.23	3.78
Anchovy	Peru	3.00	3.44	4.03
AK Pollock	US	4.88	4.28	4.12
NE Groundfish	US	2.50	2.98	3.88
AK Crab	US	4.25	4.19	4.23
