# Supplementary material for: The fishery performance indicators for global tuna fisheries
Source: Nat Commun. 2019 Apr 9;10:1641. doi: 10.1038/s41467-019-09466-6 (PMC6456575; doi:10.1038/s41467-019-09466-6)
Supplement: Supplementary file 3 — Description of Additional Supplementary Files [file 41467_2019_9466_MOESM3_ESM.pdf]

Description of Additional Supplementary Information

File Name: Supplementary Data 1

Description: FPI outcome performance and enabling condition metric scores for each aggregate tuna fishery
