# Supplementary material for: The fishery performance indicators for global tuna fisheries
Source: Nat Commun. 2019 Apr 9;10:1641. doi: 10.1038/s41467-019-09466-6 (PMC6456575; doi:10.1038/s41467-019-09466-6)
Supplement: Supplementary file 4 — Supplementary Data 1 [file 41467_2019_9466_MOESM4_ESM.pdf]

MANAGEMENT SYSTEM

SCALE OF PRODUCTION & GEAR

Indicator	Dimension	Metric	Score System	Additional Explanation
Stock Performance	Ecologically Sustainable Fisheries	Percentage of Stocks Overfished	• **5**: None overfished;<br>• **4**: 1-25% of stocks overfished;<br>• **3**: 26-50% overfished;<br>• **2**: 51-75% overfished;<br>• **1**: 76-100% overfished	Percentage of commercial stocks within the management authority's jurisdiction that are considered to be overfished, to be experiencing overfishing, or whose stock status is generally unknown. (Degree of overfishing and current trend are the next questions.) Single stock fisheries will be scored 1 or 5
		Degree of Overfishing- Stock Status	• **5**: Stock is not overfished or is rebuilt; $B/B_{MSY} \geq 1$<br>• **4**: Stock is mildly overfished ; $0.75 \leq B/B_{MSY} < 1$<br>• **3**: Stock is moderately overfished ; $0.5 \leq B/B_{MSY} < 0.75$<br>• **2**: Stock is seriously overfished; $0.25 \leq B/B_{MSY} < 0.5$<br>• **1**: Stock is severely overfished and in danger of collapse; $0.25 > B/B_{MSY}$	Current status of stock. For multispecies fisheries do a value-weighted average of the top three stocks. Two alternate scoring systems are offered. The first is for fisheries where stock levels are not known with any precision. The second is for fisheries where scientific stock assessments are being conducted and measures of B (biomass level) and BMSY
		Stock Declining, Stable or Rebuilding - Stock Dynamics	**Add or subtract from previous metric according to:**<br>• **+2:** Stock is rapidly rebuilding<br>• **+1:** Stock is rebuilding<br>• **+0:** Stock is stable<br>• **-1:** Stock is declining<br>• **-2:** Stock is rapidly declining	Extent to which current effort levels affect stock status. For multispecies fisheries do a value-weighted average of the top three stocks. This measure is scored by taking the fishery's score in the previous question and then adding or subtracting points depending on whether the fishery is rebuilding or declining. The maximum score for this measure is a 5 and the minimum score is a 1. If the fishery scored a 5 in the previous question then the score here is automatically a 5. If the fishery scored less than
		Regulatory Mortality	• **5**: No regulatory mortality of the target species;<br>• **4**: Regulatory mortality is less than 5% of total catch<br>• **3**: 5-25%<br>• **2**: 25-50%<br>• **1**: For every 100lbs of fish caught, more than 50lbs are discarded	Ratio of estimated regulatory mortality to actual landings of the target species. Regulatory mortality is defined as fish loss that is induced by regulation, such as size restrictions.
		Selectivity	• **5**: There is virtually no non-target catch<br>• **4**: Less than 5% of catch is of non-target species<br>• **3**: 5-25%<br>• **2**: 25-50%<br>• **1**: For every 100lbs of fish caught, more than 50lbs are non-target species	Percentage of total catch that is made up of non-target species. Note that non-target species are distinct from multispecies fisheries in that the catch of non-target species does not increase the value of fishing, or imposes costs on the target fishery.
		Illegal, Unregulated or Unreported Landings	• **5**: There is virtually no IUU catch<br>• **4**: Less than 5% of catch is IUU<br>• **3**: 5-25%<br>• **2**: 25-50%<br>• **1**: For every 100lbs of fish caught, more than 50lbs are IUU	Proportion of landings from the managed stock using illegal gear, area, methods, etc., or that goes unreported or falls outside of the regulatory structure. See manual for how to deal with incursion from the recreational sector.
		Status of Critical Habitat	• **5**: Critical habitat is healthy and not threatened;<br>• **4**: Less than 25% is degraded or dysfunctional;<br>• **3**: 25-75% is degraded or dysfunctional;<br>• **2**: More than 75% of critical habitat is destroyed;<br>• **1**: Nearly all critical habitat is damaged or dysfunctional	Portion of critical habitat that is damaged or dysfunctional. Critical habitat is defined as that playing a significant role in the life cycle of the fish. Portion damaged is based on area, and from all sources of damage including fishing damage, pollution and development.
		Proportion of Harvest with a 3rd Party Certification	• **5**: 76-100% of landings are certified;<br>• **4**: 51-75% of landings are certified;<br>• **3**: 26-50% of landings are certified;<br>• **2**: 1-25% of landings are certified;<br>• **1**: No landings have third party certification	The proportion of harvest (quantity) harvested under one of the recognized third party programs that certify ecological sustainability, such as the Marine Stewardship Council (MSC) certification. See manual for how to deal with other certification or fishery improvement programs
	Harvest Performance	Landings Level	• **5**: Harvest is less than MSY (stock is above MSY level) to increase profit;<br>• **4**: Harvest is approximately at MSY;<br>• **3**: Harvest reduced to promote recovery;<br>• **2**: Harvest is constraining stock recovery;<br>• **1**: Harvest is causing overfishing (stock is below MSY and declining)	Average annual harvest over the last three years. Note that this measure refers to MSY but there are many fisheries where the lack of stock assessments and reliable data mean that these estimates are unattainable. In such fisheries, score is based on discerning the goal of management/harvesters when deciding how much to land.
		Excess Capacity	• **5**: Within 5% of days required; no evidence of excess capacity<br>• **4**: 90-95%;<br>• **3**: 75-90%;<br>• **2**: 50-75%;<br>• **1**: Less than 50%, of days required; excess capacity imposes heavy costs in the fishery	In the absence of a fishery-specific measure of overfishing, use estimated standardized vessel-days required to catch the maximum sustainable yield (MSY) compared to the number of standardized vessel-days available. Days are considered not to be restricted by trip limits.
		Season Length	• **5**: Virtually no regulatory closures;<br>• **4**: 90-99%;<br>• **3**: 50-90%;<br>• **2**: 10-50%;<br>• **1**: Less than 10%	Ratio of number of days on which fishing occurs to the number of days the species is available in economically feasible quantities. This is primarily a measure of the extent of derby (including short regulatory seasons to limit total effort), not lack of biological availability or closures to prevent within-season growth overfishing

Metric	IOTC (n=7;N=4)				IATTC (n=4;N=4)				WCPFC (n=14;N=8)								ICCAT (n=4;N=3)			CCSBT (n=2;N=2)		
	DW-PS-SKJ (n=1; A)	SE-PL-SKJ (n=1; A)	SE-GNLL-SKJmsp (n=1; C)	SE-LL/HL-YFTmsp (n=4; B)	I-PS-SKJ (n=1; B)	A-YFT/BETmsp (n=1; B)	I-PLTR-ALB (n=1; A)	I-PS-BFT (n=1; A)	PNA:FSMA-I-PS-SKJ (n=, 3*; A)	I-PS-SKJ (n=1; B)	USTr-DW-PS-SKJ (n=2; A)	DW-PS-SKJ (n=2; B)	DW-LL-ALLmsp (n=2; A)	I-LL-ALLmsp (n=2; A)	SE-LL-YFT (n=1; B)	A-LL-YFT/msp (n=1; B)	DW-PS-SKJ (n=1; A)	DW-LL-ALLmsp (n=2; A)	I-PS-BFT (n=1; A)	DW-LL-ALLmsp (n=1; A)	I-PS-SBF (n=1; A)	PNA:FSMA-SE-PS-SKJ (n=2; A)
	Aggregate Score	Aggregate Score	Aggregate Score	Aggregate Score	Aggregate Score	Aggregate Score	Aggregate Score	Aggregate Score	Aggregate Score	Aggregate Score	Aggregate Score	Aggregate Score	Aggregate Score	Aggregate Score	Aggregate Score	Aggregate Score	Aggregate Score	Aggregate Score	Aggregate Score	Aggregate Score	Aggregate Score	Aggregate Score
Percentage of Stocks Overfished	5.0	5.0	5.0	5.0	3.0	3.0	5.0	1.0	5.0	3.0	3.0	3.7	3.0	3.0	4.0	3.0	4.0	1.0	1.0	1.0	1.0	5.0
Degree of Overfishing- Stock Status	5.0	5.0	5.0	5.0	4.0	4.0	5.0	1.0	5.0	4.0	4.0	5.0	4.0	4.0	4.0	4.0	5.0	2.0	1.0	5.0	2.0	5.0
Stock Declining, Stable or Rebuilding - Stock Dynamics	5.0	5.0	5.0	5.0	5.0	3.0	5.0	2.0	5.0	4.0	4.0	4.4	2.0	2.0	5.0	4.0	5.0	2.0	1.0	5.0	2.0	5.0
Regulatory Mortality	5.0	5.0	5.0	5.0	5.0	5.0	5.0	5.0	5.0	5.0	5.0	5.0	5.0	5.0	5.0	5.0	3.0	5.0	4.0	5.0	5.0	5.0
Selectivity	3.0	4.0	5.0	4.3	4.0	4.0	5.0	4.0	3.0	3.0	3.0	3.0	4.0	4.0	4.0	5.0	3.0	3.0	5.0	2.0	4.0	3.0
Illegal, Unregulated or Unreported Landings	3.0	4.0	2.0	3.0	4.0	3.0	5.0	3.0	3.0	2.0	3.0	3.0	2.0	1.0	3.0	1.0	3.0	1.0	1.0	1.0	3.0	3.0
Status of Critical Habitat	5.0	5.0	5.0	5.0	5.0	5.0	5.0	5.0	5.0	5.0	5.0	5.0	5.0	5.0	5.0	5.0	5.0	5.0	4.0	5.0	5.0	4.0
Proportion of Harvest with a 3rd Party Certification	1.0	5.0	1.0	1.0	1.0	1.0	5.0	1.0	4.0	1.0	2.0	2.0	1.0	1.0	4.0	1.0	1.0	1.0	1.0	1.0	1.0	4.0
Landings Level	4.0	4.0	4.0	4.0	3.0	3.0	4.0	3.0	4.0	4.0	3.0	3.4	2.0	2.3	4.0	4.0	3.0	2.0	1.0	1.0	1.0	4.0
Excess Capacity	3.0	4.0	1.0	2.0	3.0	3.0	4.0	4.0	3.0	3.0	3.0	3.0	3.0	2.0	2.0	1.0	3.0	2.0	3.0	3.0	4.0	3.0
Season Length	4.0	5.0	5.0	5.0	4.0	5.0	5.0	5.0	3.0	5.0	3.0	3.4	5.0	5.0	5.0	5.0	4.0	5.0	4.0	5.0	5.0	3.0

MANAGEMENT SYSTEM					IOTC (n=7;N=4)				IATTC (n=4;N=4)				WCPFC (n=14;N=8)								ICCAT (n=4;N=3)			CCSBT (n=2;N=2)		
SCALE OF PRODUCTION & GEAR					DW-PS-SKJ (n=1; A)	SE-PL-SKJ (n=1; A)	SE-GNLL-SKJmsp (n=1; C)	SE-LL/HL-YFTmsp (n=4; B)	I-PS-SKJ (n=1; B)	A-YFT/BETmsp (n=1; B)	I-PLTR-ALB (n=1; A)	I-PS-BFT (n=1; A)	PNA:FSMA-I-PS-SKJ (n=, 3*; A)	I-PS-SKJ (n=1; B)	USTr-DW-PS-SKJ (n=2; A)	DW-PS-SKJ (n=2; B)	DW-LL-ALLmsp (n=2; A)	I-LL-ALLmsp (n=2; A)	SE-LL-YFT (n=1; B)	A-LL-YFT/msp (n=1; B)	DW-PS-SKJ (n=1; A)	DW-LL-ALLmsp (n=2; A)	I-PS-BFT (n=1; A)	DW-LL-ALLmsp (n=1; A)	I-PS-SBF (n=1; A)	PNA:FSMA-SE-PS-SKJ (n=2; A)
Indicator	Dimension	Metric	Score System	Additional Explanation	Aggregate Score	Aggregate Score	Aggregate Score	Aggregate Score	Aggregate Score	Aggregate Score	Aggregate Score	Aggregate Score	Aggregate Score	Aggregate Score	Aggregate Score	Aggregate Score	Aggregate Score	Aggregate Score	Aggregate Score	Aggregate Score	Aggregate Score	Aggregate Score	Aggregate Score	Aggregate Score	Aggregate Score	Aggregate Score
		Percentage of Stocks Overfished	• 5: None overfished;<br>• 4: 1-25% of stocks overfished;<br>• 3: 26-50% overfished;<br>• 2: 51-75% overfished;<br>• 1: 76-100% overfished	Percentage of commercial stocks within the management authority's jurisdiction that are considered to be overfished, to be experiencing overfishing, or whose stock status is generally unknown. (Degree of overfishing and current trend are the next questions.) Single stock [illegible]	5.0	5.0	5.0	5.0	3.0	3.0	5.0	1.0	5.0	3.0	3.0	3.7	3.0	3.0	4.0	3.0	4.0	1.0	1.0	1.0	1.0	5.0
		Harvest Safety	• 5: Less than 0.1 deaths per thousand person seasons;<br>• 4: Less than 0.5 deaths;<br>• 3: Less than 1;<br>• 2: Less than 5<br>• 1: More than 5 deaths per thousand person seasons	Number of harvester (captain or crew) on-the-job deaths, per thousand person fishing season. We consider there to be one season per year, but do not annualize mortality if the fishing season is less than a year.	5.0	5.0	2.0	3.3	3.0	4.0	5.0	5.0	3.0	3.0	3.7	3.7	3.5	3.6	4.0	3.0	4.0	3.0	5.0	1.0	5.0	4.0
	Harvest Asset Performance	Ratio of Asset Value to Gross Earnings	• 5: 10 or higher;<br>• 4: 7.5-10;<br>• 3: 5-7.5;<br>• 2: 2.5-5;<br>• 1: Below 2.5	Extent to which fishery wealth is accumulated in access capital (e.g., quota, permits or vessels). Ratio of average price of capital and licenses required to access the fishery over the last five years to the average annual gross earnings for a similarly scaled access right in the same period. Typically a 1 if vessels or quota not limited by regulation. Same business or same family sales are excluded, where they can be identified. See 'Historical Data' tab.	1.0	1.0	1.0	1.0	1.0	1.0	1.0	1.0	1.0	1.0	1.0	1.0	1.0	1.0	1.0	1.0	1.0	1.0	1.0	1.0	1.0	1.0
		Total Revenue Compared to Historic High	• 5: Above 95%;<br>• 4: 85-95%;<br>• 3: 70-85%;<br>• 2: 50-70%;<br>• 1: Below 50%	The indicator is the ratio of total real revenue (in local currency) to the average of the three highest total real revenues in the past 10 years. Adjust by local CPI if inflation was significant. See 'Historical Data' Tab.	2.0	5.0	4.0	4.3	3.0	3.0	5.0	5.0	3.0	4.0	4.3	5.0	4.3	4.4	1.0	5.0	3.0	4.0	5.0	2.0	5.0	1.7
		Asset (Permit, Quota, etc...) Value Compared to Historic High	• 5: Above 95%;<br>• 4: 85-95%;<br>• 3: 70-85%;<br>• 2: 50-70%;<br>• 1: Below 50%	The indicator is the ratio of the current value of the harvest asset (permit, quota, vessel, etc...) to the average of the three highest asset values in the past 10 years. Adjust by local CPI if inflation was significant. Typically 1 if wealth is not accumulating in vessels, permits or quota. See 'Historical Data' tab.	3.0	5.0	5.0	5.0	5.0	5.0	5.0	5.0	5.0	5.0	1.0	2.0	2.0	2.0	5.0	5.0	3.0	3.0	5.0	3.0	5.0	5.0
		Borrowing Rate Compared to Risk-free Rate	• 5: Less than 1.75; cf. 30-year conforming mortgage;<br>• 4: Less than 2.5; cf. personal bank loan;<br>• 3: Less than 4; cf. good credit card rates;<br>• 2: Less than 7; cf. bad credit card rates;<br>• 1: Greater than 7; usury	Average ratio between the interest rate on loans made to harvesters in the industry to risk-free rates over the last three years. If businesses can access international credit markets, then the international risk free rate (US 10 year Treasury Bill) is an appropriate comparison; [illegible]	5.0	4.0	5.0	3.3	5.0	1.0	5.0	5.0	3.0	1.0	5.0	5.0	5.0	5.0	4.0	5.0	4.0	5.0	5.0	5.0	5.0	3.6
		Source of Capital	• 5: Unsecured business loans from banks/Venture capital;<br>• 4: Secured business loans from banks/Public stock offering; investment from elsewhere in supply chain<br>• 3: Loans from banks secured by personal (not business) assets/Government subsidized private lending/Government-run loan programs/International aid agencies; secured loans from elsewhere in supply chain<br>• 2: Microlending/Family/community-based lending; loans from supply chain significantly reduce margins<br>• 1: Mafia/No capital available; exploitative relationship from elsewhere in [illegible]	Points to be assigned based on the category of lenders or investors that are most typically used by harvesters in the fishery. Second scoring method offered if the supply chain (e.g., traders, processors, exporters) are the primary source of capital.	5.0	4.0	3.0	3.6	4.0	1.0	5.0	5.0	5.0	4.0	3.6	5.0	5.0	5.0	4.0	4.0	4.0	5.0	5.0	5.0	5.0	2.0
		Functionality of Harvest Capital	• 5: Capital is new;<br>• 4: Capital is older but well maintained, e.g., freshly painted;<br>• 3: Capital is moderately well maintained;<br>• 2: Maintenance is poor;<br>• 1: Serious concerns about seaworthiness or safety throughout fishery	Average age of the key durable harvesting capital unit (vessels, weirs). Ages are not assigned to scores due to differences in expected useful life, but buildings and industrial vessels have expected life of roughly 20 years.	5.0	5.0	2.0	3.0	4.0	2.0	4.0	4.0	3.0	3.0	4.3	4.6	3.0	3.7	3.0	2.0	5.0	3.3	4.0	4.0	4.0	4.0
		Annual Total Revenue Volatility	• 5: Less than 0.15<br>• 4: 0.15-0.22;<br>• 3: 0.22-0.40;<br>• 2: 0.40-1;<br>• 1: Greater than 1	Ratio of the standard deviation of the first differences of annual total revenue to the mean of total revenue over the last 10 years. Best guess may be calculated based on shorter time series if data not available. See 'Volatility' Tab.	3.0	2.0	4.0	2.8	2.0	1.0	3.0	3.0	3.0	3.0	3.0	3.0	3.5	3.4	2.0	2.0	3.0	4.0	3.0	2.0	3.0	2.3
		Annual Landings Volatility	• 5: Less than 0.15<br>• 4: 0.15-0.22;<br>• 3: 0.22-0.40;<br>• 2: 0.40-1;<br>• 1: Greater than 1	Ratio of the standard deviation of the first differences of annual total landings to the mean of total landings over the last 10 years. Best guess may be calculated based on shorter time series if data not available. See 'Volatility' tab.	4.0	2.0	3.0	2.4	3.0	3.0	4.0	4.0	4.0	3.0	4.0	4.0	4.8	4.7	3.0	3.0	4.0	5.0	4.0	4.0	4.0	3.0
		Intra-annual Landings Volatility	• 5: Less than 0.15<br>• 4: 0.15-0.22;<br>• 3: 0.22-0.40;<br>• 2: 0.40-1;<br>• 1: Greater than 1	Ratio of the standard deviation of the weekly/monthly total landings over the last three years to the mean of total landings. Observations of zero landings are included if there is biological availability. If the biological season is so short that there is not meaningful variation at a monthly level, this measure can be NA. Best guess may be calculated based on shorter time series if data not available. See 'Volatility' tab.	3.0	2.0	4.0	1.0	5.0	4.0	1.0	1.0	3.0	3.0	3.0	3.0	3.0	3.0	1.0	1.0	3.0	3.0	1.0	2.0	1.0	3.0

MANAGEMENT SYSTEM

SCALE OF PRODUCTION & GEAR

Indicator	Dimension	Metric	Score System	Additional Explanation
Harvest Sector Performance	Risks	Percentage of Stocks Overfished	• **5**: None overfished;<br>• **4**: 1-25% of stocks overfished;<br>• **3**: 26-50% overfished;<br>• **2**: 51-75% overfished;<br>• **1**: 76-100% overfished	Percentage of commercial stocks within the management authority's jurisdiction that are considered to be overfished, to be experiencing overfishing, or whose stock status is generally unknown. (Degree of overfishing and current trend are the next questions.) Single stock [illegible]
		Annual Price Volatility	• **5**: Less than 0.13;<br>• **4**: 0.13-0.20;<br>• **3**: 0.20-0.30;<br>• **2**: 0.30-0.85;<br>• **1**: Greater than 0.85	Ratio of the standard deviation of the first differences of annual ex-vessel prices to the mean of ex-vessel price over the last 10 years. Best guess may be calculated based on shorter time series if data not available. See 'Volatility' tab.
		Intra-annual Price Volatility	• **5**: Less than 0.13;<br>• **4**: 0.13-0.20;<br>• **3**: 0.20-0.30;<br>• **2**: 0.30-0.85;<br>• **1**: Greater than 0.85	Ratio of the standard deviation of average monthly ex-vessel prices over the last three years to the mean ex-vessel price. Observations of zero landings are included if there is biological availability. If the biological season is so short that there is not meaningful variation at a monthly level, this measure can be NA. Best guess may be calculated based on shorter time series if data not available. See 'Volatility' tab.
		Spatial Price Volatility	• **5**: Less than 0.13;<br>• **4**: 0.13-0.20;<br>• **3**: 0.20-0.30;<br>• **2**: 0.30-0.85;<br>• **1**: Greater than 0.85	Ratio of the standard deviation across data collection regions of average annual ex-vessel price to the mean of ex-vessel price across data collection regions. Measure should be averaged over last three years. Best guess may be calculated based on shorter time series if data [illegible]
		Contestability & Legal Challenges	• **5**: No significant legal challenges, civil actions, or protests regarding the fishery management system;<br>• **4**: Minor legal challenges slow implementation;<br>• **3**: Legal challenges, civil actions, or protests impede some management measures;<br>• **2**: Legal challenges, civil actions, or protests suspend major elements of the management system;<br>• **1**: Legal challenges, civil actions, or protests suspend or prohibit [illegible]	This captures the degree to which political activity limits the ability to implement effective fishing regulations.
	Owners, Permit Holders & Captains (Those holding the right or ability to access)	Earnings Compared to Regional Average Earnings	• **5**: More than 50% above the regional average;<br>• **4**: Between 10 and 50% above the regional average;<br>• **3**: Within 10% of the regional average;<br>• **2**: Between 50% and 90% of the regional average;<br>• **1**: Less than half of the regional average	Ratio of annual earnings per owner/captain to the regional average earnings. In many cases, the captain is an owner of a vessel or permit, but in other cases, captains are considered as crew. The owners are defined as those holding the ability to access, including rights and [illegible]
		Owner/Permit Holder/Captain Wages Compared to Non-fishery Wages	• **5**: More than 50% above the alternative wage;<br>• **4**: Between 10 and 50% above the alternative wage;<br>• **3**: Within 10% of the alternative wage;<br>• **2**: Between 50% and 90% of the alternative wage;<br>• **1**: Less than half of the alternative wage	Ratio of captain's average daily wage in this fishery to the average daily wage in the owner/captain's alternate occupations within their economic sphere. The comparison is to jobs in the village that the owner/captain qualifies for if all economic activity is within the village, but to jobs within the nation if the owner/captain participates in national markets [illegible]
		Education Access	• **5**: Higher education is accessible;<br>• **4**: High school level education or advanced technical training is accessible;<br>• **3**: Middle school level education or simple technical training is accessible;<br>• **2**: Basic literacy and arithmetic training is accessible;<br>• **1**: Formal education is not accessible	Measure is based on the highest level of education that is accessible to (available and affordable) the families (i.e., children) of permit holders and captains. Not based on the actual educational attainment levels of current captains and owners.
		Access to Health Care	• **5**: Global standard treatment for illness is accessible;<br>• **4**: Licensed doctors provide trauma, surgical and drug treatments;<br>• **3**: Nurses or medical practitioners provide emergency and routine drug treatments;<br>• **2**: Basic and simple drug treatment is accessible;<br>• **1**: Medical or drug treatment is not accessible	Measure is based on the quality of health care that is accessible to (available and affordable) the owners/permit holders and their families.
		Social Standing of Boat Owners and Permit Holders	• **5**: Among the most respected in the community, comparable with civic and religious leaders and professionals, such as doctors and lawyers;<br>• **4**: Comparable to management and white collar jobs;<br>• **3**: Comparable to skilled labor jobs;<br>• **2**: Comparable to unskilled blue collar or service jobs;<br>• **1**: Among the least respected, such as slaves or indentured servants	Measure is based on the social standing of owners/permit holders/captains within the community where they spend the majority of their time.
		Proportion of Nonresident Employment	• **5**: 95-100% local;<br>• **4**: 70-95% local;<br>• **3**: 35-70% local;<br>• **2**: 5-35% local;<br>• **1**: Virtually no local captains/permit holders	Proportion of owners/permit holders/captains who are local. "Local" is defined as coming from, and spending their earnings within, the local fishing community. Nationals who are transient nonresidents, or considered outsiders in the fishing community, are not local.

Aggregate Score:

Metric	IOTC (n=7;N=4)				IATTC (n=4;N=4)				WCPFC (n=14;N=8)								ICCAT (n=4;N=3)			CCSBT (n=2;N=2)		
	DW-PS-SKJ (n=1; A)	SE-PL-SKJ (n=1; A)	SE-GNLL-SKJmsp (n=1; C)	SE-LL/HL-YFTmsp (n=4; B)	I-PS-SKJ (n=1; B)	A-YFT/BETmsp (n=1; B)	I-PLTR-ALB (n=1; A)	I-PS-BFT (n=1; A)	PNA:FSMA-I-PS-SKJ (n=, 3*; A)	I-PS-SKJ (n=1; B)	USTr-DW-PS-SKJ (n=2; A)	DW-PS-SKJ (n=2; B)	DW-LL-ALLmsp (n=2; A)	I-LL-ALLmsp (n=2; A)	SE-LL-YFT (n=1; B)	A-LL-YFT/msp (n=1; B)	DW-PS-SKJ (n=1; A)	DW-LL-ALLmsp (n=2; A)	I-PS-BFT (n=1; A)	DW-LL-ALLmsp (n=1; A)	I-PS-SBF (n=1; A)	PNA:FSMA-SE-PS-SKJ (n=2; A)
Percentage of Stocks Overfished	5.0	5.0	5.0	5.0	3.0	3.0	5.0	1.0	5.0	3.0	3.0	3.7	3.0	3.0	4.0	3.0	4.0	1.0	1.0	1.0	1.0	5.0
Annual Price Volatility	4.0	3.0	4.0	2.4	2.0	2.0	2.0	2.0	3.0	2.0	2.0	2.4	1.8	1.7	2.0	2.0	4.0	2.0	2.0	2.0	2.0	1.6
Intra-annual Price Volatility	4.0	3.0	2.0	2.4	4.0	3.0	3.0	2.0	2.0	3.0	3.0	3.4	1.8	1.7	2.0	2.0	4.0	2.0	2.0	2.0	2.0	1.3
Spatial Price Volatility	5.0	4.0	2.0	3.0	5.0	4.0	4.0	3.0	5.0	5.0	5.0	5.0	5.0	5.0	5.0	2.0	5.0	5.0	5.0	3.0	5.0	5.0
Contestability & Legal Challenges	5.0	5.0	3.0	3.0	4.0	5.0	5.0	5.0	3.0	5.0	4.0	5.0	5.0	5.0	5.0	5.0	5.0	5.0	2.0	2.0	2.0	3.0
Earnings Compared to Regional Average Earnings	5.0	5.0	5.0	5.0	5.0	3.0	5.0	5.0	5.0	5.0	5.0	5.0	5.0	5.0	3.0	5.0	5.0	5.0	5.0	5.0	5.0	5.0
Owner/Permit Holder/Captain Wages Compared to Non-fishery Wages	5.0	5.0	4.0	4.6	5.0	3.0	4.0	5.0	5.0	5.0	4.0	4.6	4.8	4.7	2.0	5.0	5.0	5.0	4.0	4.0	5.0	5.0
Education Access	5.0	4.0	4.0	4.0	5.0	3.0	5.0	5.0	3.0	4.0	5.0	5.0	5.0	5.0	4.0	3.0	5.0	5.0	5.0	5.0	5.0	3.7
Access to Health Care	5.0	3.0	4.0	3.8	4.0	4.0	5.0	4.0	3.0	4.0	4.4	4.4	4.3	4.3	4.0	2.0	5.0	4.0	5.0	5.0	5.0	3.4
Social Standing of Boat Owners and Permit Holders	5.0	5.0	3.0	3.6	5.0	2.0	4.0	5.0	4.0	3.0	4.0	4.4	4.3	4.3	4.0	3.0	5.0	4.0	5.0	5.0	5.0	4.3
Proportion of Nonresident Employment	5.0	5.0	5.0	5.0	5.0	5.0	5.0	5.0	3.0	5.0	3.1	5.0	5.0	5.0	2.0	5.0	5.0	5.0	5.0	5.0	5.0	3.3

MANAGEMENT SYSTEM					IOTC (n=7;N=4)				IATTC (n=4;N=4)				WCPFC (n=14;N=8)								ICCAT (n=4;N=3)			CCSBT (n=2;N=2)		
SCALE OF PRODUCTION & GEAR					DW-PS-SKJ (n=1; A)	SE-PL-SKJ (n=1; A)	SE-GNLL-SKJmsp (n=1; C)	SE-LL/HL-YFTmsp (n=4; B)	I-PS-SKJ (n=1; B)	A-YFT/BETmsp (n=1; B)	I-PLTR-ALB (n=1; A)	I-PS-BFT (n=1; A)	PNA:FSMA-I-PS-SKJ (n=, 3*; A)	I-PS-SKJ (n=1; B)	USTr-DW-PS-SKJ (n=2; A)	DW-PS-SKJ (n=2; B)	DW-LL-ALLmsp (n=2; A)	I-LL-ALLmsp (n=2; A)	SE-LL-YFT (n=1; B)	A-LL-YFT/msp (n=1; B)	DW-PS-SKJ (n=1; A)	DW-LL-ALLmsp (n=2; A)	I-PS-BFT (n=1; A)	DW-LL-ALLmsp (n=1; A)	I-PS-SBF (n=1; A)	PNA:FSMA-SE-PS-SKJ (n=2; A)
Indicator	Dimension	Metric	Score System	Additional Explanation	Aggregate Score	Aggregate Score	Aggregate Score	Aggregate Score	Aggregate Score	Aggregate Score	Aggregate Score	Aggregate Score	Aggregate Score	Aggregate Score	Aggregate Score	Aggregate Score	Aggregate Score	Aggregate Score	Aggregate Score	Aggregate Score	Aggregate Score	Aggregate Score	Aggregate Score	Aggregate Score	Aggregate Score	Aggregate Score
		Percentage of Stocks Overfished	• 5: None overfished;<br>• 4: 1-25% of stocks overfished;<br>• 3: 26-50% overfished;<br>• 2: 51-75% overfished;<br>• 1: 76-100% overfished	Percentage of commercial stocks within the management authority's jurisdiction that are considered to be overfished, to be experiencing overfishing, or whose stock status is generally unknown. (Degree of overfishing and current trend are the next questions.) Single stock [illegible]	5.0	5.0	5.0	5.0	3.0	3.0	5.0	1.0	5.0	3.0	3.0	3.7	3.0	3.0	4.0	3.0	4.0	1.0	1.0	1.0	1.0	5.0
	Crew (Those depending on others for access)	Earnings Compared to Regional Average Earnings	• 5: More than 50% above the regional average;<br>• 4: Between 10 and 50% above regional average;<br>• 3: Within 10% of the regional average;<br>• 2: Between 50% and 90% of the regional average;<br>• 1: Less than half of the regional average	Ratio of annual earnings per crew member to the regional average earnings. In many cases, the captain is an owner of a vessel or permit, but in other cases, captains are considered as crew. Crew is defined as those depending on others for access. Note that this is earnings	5.0	5.0	1.0	4.8	4.0	1.0	4.0	5.0	3.0	3.0	5.0	5.0	4.3	4.3	3.0	5.0	5.0	4.0	4.0	5.0	3.0	4.0
		Crew Wages Compared to Non-fishery Wages	• 5: More than 50% above the alternative wage;<br>• 4: Between 10 and 50% above alternative wage;<br>• 3: Within 10% of the alternative wage;<br>• 2: Between 50% and 90% of the alternative wage;<br>• 1: Less than half of the alternative wage	Ratio of crew's average daily wage in this fishery to the average daily wage in the crew's alternate occupations within their economic sphere. The comparison is to jobs in the village that the crew qualifies for if all economic activity is within the village, but to jobs within the nation if the crew participates in national markets as a consumer and labor markets are fluid. Meant to capture the average personal opportunity cost of participating in the fishery.	5.0	5.0	2.0	5.0	4.0	1.0	3.0	5.0	3.0	4.0	5.0	5.0	4.3	4.3	2.0	4.0	5.0	4.0	4.0	5.0	2.0	3.0
		Education Access	• 5: Higher education is accessible;<br>• 4: High school level education or advanced technical training is accessible;<br>• 3: Middle school level education or simple technical training is accessible;<br>• 2: Basic literacy and arithmetic training is accessible;<br>• 1: Formal education is not accessible	Measure is based on the highest level of education that is accessible to (available and affordable) the families (i.e., children) of crew. Not based on the actual educational attainment levels of current crew members.	3.0	3.0	3.0	3.0	4.0	3.0	5.0	5.0	2.0	3.0	4.0	4.0	3.3	3.3	4.0	3.0	3.0	3.0	5.0	4.0	4.0	2.7
		Access to Health Care	• 5: Global standard treatment for illness is accessible;<br>• 4: Licensed doctors provide trauma, surgical and drug treatments;<br>• 3: Nurses or medical practitioners provide emergency and routine drug treatments;<br>• 2: Basic and simple drug treatment is accessible;<br>• 1: Medical or drug treatment is not accessible	Measure is based on the quality of health care that is accessible to (available and affordable) the crew and their families.	3.0	3.0	4.0	3.8	4.0	4.0	5.0	4.0	2.0	4.0	4.0	3.4	3.0	3.0	4.0	2.0	3.0	3.0	5.0	3.0	4.0	3.4
		Social Standing of Crew	• 5: Among the most respected in the community, comparable with civic and religious leaders and professionals, such as doctors and lawyers;<br>• 4: Comparable to management and white collar jobs;<br>• 3: Comparable to skilled labor jobs;<br>• 2: Comparable to unskilled blue collar or service jobs;<br>• 1: Among the least respected, such as slaves or indentured servants	Measure is based on the social standing of crew members within the community where they spend the majority of their time.	5.0	3.0	2.0	2.6	3.0	1.0	3.0	4.0	3.0	3.0	3.0	3.0	2.0	2.3	3.0	3.0	5.0	2.0	4.0	2.0	2.0	3.3
		Proportion of Nonresident Employment	• 5: 95-100% local;<br>• 4: 70-95% local;<br>• 3: 35-70% local;<br>• 2: 5-35% local;<br>• 1: Virtually no local crew	Proportion of crew members who are local. "Local" is defined as coming from, and spending their earnings within, the local fishing community. Nationals who are transient nonresidents, or considered outsiders in the fishing community, are not local.	2.0	5.0	4.0	4.4	5.0	5.0	5.0	5.0	3.0	5.0	1.4	2.6	1.8	1.7	3.0	5.0	2.0	2.0	4.0	1.0	5.0	4.0
		Crew Experience	• 5: More than 10 years (skilled career crew);<br>• 4: 5-10 years;<br>• 3: 3-5 years;<br>• 2: 1-3 years;<br>• 1: 0 full years of experience (mostly new crew each season)	Average years of experience of crew members.	5.0	4.0	5.0	4.6	5.0	4.0	5.0	4.0	5.0	3.0	4.0	4.0	3.8	3.7	4.0	5.0	5.0	4.0	4.0	3.0	4.0	4.3
		Age Structure of Harvesters	• 5: All working ages are well represented;<br>• 4: Slightly skewed toward younger or older;<br>• 3: Skewed toward younger or older;<br>• 2: Almost entirely younger or older, but working age;<br>• 1: Harvesters primarily younger or older than working age	Age range of both captains and their crews.	5.0	4.0	4.0	4.4	4.0	5.0	3.0	3.0	3.0	5.0	3.0	3.0	2.8	2.7	3.0	4.0	5.0	3.0	4.0	2.0	4.0	3.0
		Ex-vessel Price Compared to Historic High	• 5: Above 95%;<br>• 4: 85-95%;<br>• 3: 70-85%;<br>• 2: 50-70%;<br>• 1: Below 50%	The indicator is the ratio of current ex-vessel prices to the average of the three highest annual ex-vessel prices in the past 10 years. Adjust by local CPI if inflation was significant. See 'Historical Data' tab.	5.0	5.0	5.0	4.4	5.0	5.0	5.0	4.0	5.0	5.0	4.6	4.3	5.0	4.0	5.0	5.0	5.0	4.7	4.0	5.0	4.0	5.0
		Final Market Use	• 5: Premium human consumption (premium quality and products);<br>• 4: High-value human consumption;<br>• 3: Moderate-value human consumption;<br>• 2: Low-value human consumption;<br>• 1: Fish meal/animal feed/bait or non-consumptive	The measure indicates the final market use of the top three species. Where a supply chain is diverse, score each and weight by value. Premium products are typically distinct to species, or species and source.	3.0	3.0	2.0	4.3	3.0	4.0	3.0	5.0	2.0	3.0	2.0	3.7	4.3	5.0	5.0	4.0	3.0	4.3	5.0	5.0	5.0	3.0
		International Trade	• 5: 90-100% export;<br>• 4: 60-90% export;<br>• 3: 30-60% export;<br>• 2: 2-30% export;<br>• 1: Virtually no export	Percentage of the fishery's value that is from fish exported to higher value international markets for consumption	5.0	4.0	1.0	4.6	3.0	4.0	4.0	5.0	4.0	2.0	3.9	3.9	4.0	3.8	5.0	5.0	5.0	5.0	5.0	3.0	5.0	5.0

MANAGEMENT SYSTEM

SCALE OF PRODUCTION & GEAR

Indicator	Dimension	Metric	Score System	Additional Explanation
	Market Performance	Percentage of Stocks Overfished	• **5**: None overfished;<br>• **4**: 1-25% of stocks overfished;<br>• **3**: 26-50% overfished;<br>• **2**: 51-75% overfished;<br>• **1**: 76-100% overfished	Percentage of commercial stocks within the management authority's jurisdiction that are considered to be overfished, to be experiencing overfishing, or whose stock status is generally unknown. (Degree of overfishing and current trend are the next questions.) Single stock fisheries will be scored 1 or 5.
		Final Market Wealth	• **5**: Greater than 35,000USD;<br>• **4**: Greater than 25,000USD;<br>• **3**: Greater than 12,500USD;<br>• **2**: Greater than 5,000USD;<br>• **1**: Less than 5,000USD	Average per capita GDP of the consumer of a fishery's primary final product. If multiple important products, weight by value.
		Wholesale Price Compared to Similar Products	• **5**: More than twice global average;<br>• **4**: 120-200% of global average;<br>• **3**: Within 20% of global average;<br>• **2**: 50-80% of global average;<br>• **1**: Less than half global average	Ratio of average price for wholesale fish product from the fishery, to the global average price for similar species. Convert the price of fish to global currency for comparison (i.e. make sure that both prices are in USD when
		Capacity of Firms to Export to the US & EU	• **5**: Over 90% meet US and EU health and labeling standards<br>• **4**: 50-90%;<br>• **3**: Less than 50%;<br>• **2**: A small amount of product meets US/EU standards;<br>• **1**: Banned in the US or EU, or cost of compliance with US/EU standards is prohibitive	Percentage of a country's fish exports that meet US or EU health and labeling standards. This is usually a country level measure, though individual high-value fisheries sometimes develop their own supply chains; measure refers to all processing capacity for export, including to regional markets.
		Ex-vessel to Wholesale Marketing Margins	• **5**: More than 200% increase in value;<br>• **4**: 100-200%;<br>• **3**: 50-100%;<br>• **2**: 10-50%;<br>• **1**: Less than 10% increase in value	Increase in value of processed wholesale product from unprocessed ex-vessel product. [(Wholesale $/lb.) - Ex Vessel $/lb.]/(Ex Vessel $/lb.)
	Post-harvest, Processing & Support Industry Performance	Processing Yield	• **5**: At feasible frontier;<br>• **4**: Within 5% of the feasible frontier;<br>• **3**: Within 10% ;<br>• **2**: Within 25%;<br>• **1**: Less than 75% of maximum yield	Ratio of actual processing yield (kilos/pounds) to the maximum processing yield technically achievable.
		Shrink	• **5**: Less than 5%<br>• **4**: 5-10%<br>• **3**: 10-25%;<br>• **2**: 25-50%<br>• **1**: More than 50%	Percentage of fishery product weight that is lost due to handling, spoilage, or theft. This is very likely to be an estimate.
		Capacity Utilization Rate	• **5**: Virtually year-round;<br>• **4**: 75-95% of days;<br>• **3**: 50-75%;<br>• **2**: 20%-50%;<br>• **1**: Less than 20%	Days open for processing each year. Such days would not normally include religious or civic holidays, or weekly rest days. This should be full time employment days; when the plant is open but only operating at 10% capacity then
		Product Improvement	• **5**: 75-100% of landings are enhanced;<br>• **4**: 50-75%;<br>• **3**: 25-50%;<br>• **2**: 1-25%;<br>• **1**: No landings have enhancements	Proportion of harvest meat weight going into certified, branded, fresh premium, portioned, live or value added products.
		Sanitation	• **5**: Sanitation in landing and processing areas meets global health standards;<br>• **4**: Basic treatment, but falls short of global standards;<br>• **3**: Human waste is adequately handled, but fish waste presents sanitation issues;<br>• **2**: Functional toilets are available, but fish or fish handlers exposed to untreated sewage;<br>• **1**: Functional toilets are not available in landing or processing areas	This measures the sanitation conditions in the landing and processing areas. This measure is scored relative to global standards, not local standards. Pit latrines or toilets that are not improved, do not have proper drainage/sewage treatment, and do not allow for proper washing do not count as functional toilets.
		Regional Support Businesses	• **5**: All types of support are plentiful;<br>• **4**: Some types of support are capacity constrained or unavailable;<br>• **3**: Most types of support are capacity constrained or unavailable;<br>• **2**: Support limited to variable inputs;<br>• **1**: Industry support is not locally available	Support businesses are those that provide critical inputs (e.g. food, ice, gear, boat maintenance) or post-harvest functions (e.g. brokering, logistics).
		Borrowing Rate Compared to Risk-free Rate	• **5**: Less than 1.75; cf. 30-year conforming mortgage;<br>• **4**: Less than 2.5; cf. personal bank loan;<br>• **3**: Less than 4; cf. good credit card rates;<br>• **2**: Less than 7; cf. bad credit card rates;<br>• **1**: Greater than 7; usury	Average ratio between the interest rate on loans made in the processing industry to risk-free rates over the last three years. If businesses can access international credit markets, then the international risk free rate (US 10 year Treasury Bill) is an appropriate comparison; otherwise, use local risk free rate.

Metric	IOTC (n=7;N=4)				IATTC (n=4;N=4)				WCPFC (n=14;N=8)								ICCAT (n=4;N=3)			CCSBT (n=2;N=2)		
	DW-PS-SKJ (n=1; A)	SE-PL-SKJ (n=1; A)	SE-GNLL-SKJmsp (n=1; C)	SE-LL/HL-YFTmsp (n=4; B)	I-PS-SKJ (n=1; B)	A-YFT/BETmsp (n=1; B)	I-PLTR-ALB (n=1; A)	I-PS-BFT (n=1; A)	PNA:FSMA-I-PS-SKJ (n=, 3*; A)	I-PS-SKJ (n=1; B)	USTr-DW-PS-SKJ (n=2; A)	DW-PS-SKJ (n=2; B)	DW-LL-ALLmsp (n=2; A)	I-LL-ALLmsp (n=2; A)	SE-LL-YFT (n=1; B)	A-LL-YFT/msp (n=1; B)	DW-PS-SKJ (n=1; A)	DW-LL-ALLmsp (n=2; A)	I-PS-BFT (n=1; A)	DW-LL-ALLmsp (n=1; A)	I-PS-SBF (n=1; A)	PNA:FSMA-SE-PS-SKJ (n=2; A)
	Aggregate Score	Aggregate Score	Aggregate Score	Aggregate Score	Aggregate Score	Aggregate Score	Aggregate Score	Aggregate Score	Aggregate Score	Aggregate Score	Aggregate Score	Aggregate Score	Aggregate Score	Aggregate Score	Aggregate Score	Aggregate Score	Aggregate Score	Aggregate Score	Aggregate Score	Aggregate Score	Aggregate Score	Aggregate Score
Percentage of Stocks Overfished	5.0	5.0	5.0	5.0	3.0	3.0	5.0	1.0	5.0	3.0	3.0	3.7	3.0	3.0	4.0	3.0	4.0	1.0	1.0	1.0	1.0	5.0
Final Market Wealth	5.0	5.0	1.0	5.0	4.0	5.0	5.0	5.0	5.0	5.0	5.0	4.4	5.0	5.0	5.0	5.0	5.0	5.0	5.0	5.0	5.0	5.0
Wholesale Price Compared to Similar Products	4.0	3.0	3.0	3.3	3.0	3.0	4.0	5.0	3.0	3.0	3.0	3.7	3.0	4.0	4.0	2.0	3.0	3.3	5.0	5.0	5.0	3.0
Capacity of Firms to Export to the US & EU	5.0	5.0	1.0	5.0	5.0	5.0	5.0	5.0	5.0	5.0	5.0	5.0	5.0	5.0	5.0	5.0	5.0	5.0	5.0	5.0	5.0	5.0
Ex-vessel to Wholesale Marketing Margins	2.0	2.0	2.0	4.3	3.0	2.0	3.0	5.0	2.0	2.0	2.0	3.1	5.0	5.0	5.0	5.0	2.0	5.0	5.0	5.0	5.0	2.0
Processing Yield	4.0	4.0	5.0	3.9	5.0	4.0	5.0	5.0	5.0	4.0	4.6	5.0	5.0	5.0	4.0	3.0	4.0	5.0	5.0	5.0	5.0	3.0
Shrink	5.0	4.0	3.0	4.3	5.0	4.0	5.0	4.0	4.0	5.0	5.0	5.0	5.0	5.0	4.0	4.0	5.0	5.0	4.0	5.0	4.0	4.0
Capacity Utilization Rate	5.0	2.0	5.0	2.9	5.0	3.0	4.0	4.0	5.0	3.0	3.6	4.4	4.3	5.0	4.0	2.0	5.0	4.3	4.0	5.0	4.0	1.0
Product Improvement	5.0	2.0	1.0	5.0	5.0	5.0	5.0	5.0	5.0	4.0	2.4	2.5	5.0	5.0	5.0	5.0	4.0	5.0	5.0	5.0	5.0	2.9
Sanitation	4.0	3.0	1.0	3.3	4.0	4.0	5.0	3.0	2.0	3.0	2.7	3.1	4.3	5.0	4.0	1.0	4.0	4.3	5.0	5.0	5.0	3.4
Regional Support Businesses	4.0	3.0	3.0	3.3	4.0	4.0	5.0	5.0	1.0	4.0	4.6	5.0	5.0	5.0	3.0	2.0	4.0	4.0	5.0	5.0	5.0	3.4
Borrowing Rate Compared to Risk-free Rate	2.0	4.0	4.0	4.0	5.0	5.0	5.0	5.0	2.0	4.0	4.4	4.4	4.3	5.0	3.0	4.0	3.0	4.3	5.0	5.0	5.0	4.1

MANAGEMENT SYSTEM

SCALE OF PRODUCTION & GEAR

Indicator	Dimension	Metric	Score System	Additional Explanation
Post-Harvest Performance	Post-Harvest Asset Performance	Percentage of Stocks Overfished	• **5**: None overfished;<br>• **4**: 1-25% of stocks overfished;<br>• **3**: 26-50% overfished;<br>• **2**: 51-75% overfished;<br>• **1**: 76-100% overfished	Percentage of commercial stocks within the management authority's jurisdiction that are considered to be overfished, to be experiencing overfishing, or whose stock status is generally unknown. (Degree of overfishing and current trend are the next questions.) Single stock [illegible]
		Source of Capital	• **5**: Unsecured business loans from banks/Venture capital;<br>• **4**: Secured business loans from banks/Public stock offering; investment from elsewhere in supply chain<br>• **3**: Loans from banks secured by personal (not business) assets/Government subsidized private lending/Government-run loan programs/International aid agencies; secured loans from elsewhere in supply chain<br>• **2**: Microlending/Family/community-based lending; loans from supply chain significantly reduce margins<br>• **1**: Mafia/No capital available; exploitative relationship from elsewhere in supply chain	Points to be assigned based on the category of lenders or investors that are most typically used in the processing sector. Second scoring method offered if the supply chain (e.g., processors further up the supply chain, parent company, exporters) are primary source of capital.
		Age of Facilities	• **5**: 1st quarter of expected life; less than 7 years for a building<br>• **4**: 2nd quarter of expected life; 7-15 years;<br>• **3**: Third quarter of expected life; 16-20 years;<br>• **2**: 4th quarter of expected life; 21-25 years;<br>• **1**: Exceeding expected life; Greater than 25 years;	Average age of the key durable processing capital unit (plants, catcher-processor vessels).
	Processing Owners & Managers	Earnings Compared to Regional Average Earnings	• **5**: More than 50% above the regional average;<br>• **4**: Between 10 and 50% above regional average;<br>• **3**: Within 10% above the regional average;<br>• **2**: Between 50% and 90% of the regional average;<br>• **1**: Less than half of the regional average	Ratio of annual earnings per owner/manager to the regional average earnings. This measure can include wealth accumulated to traders/middlemen if they represent an important part of the supply chain. Note that [illegible]
		Manager Wages Compared to Non-fishery Wages	• **5**: More than 50% above the alternative wage;<br>• **4**: Between 10 and 50% above alternative wage;<br>• **3**: Within 10% above the alternative wage;<br>• **2**: Between 50% and 90% of the alternative wage;<br>• **1**: Less than half of the alternative wage	Ratio of owner/manager's average daily wage in this fishery to the average daily wage in the owner/manager's alternate occupations within their economic sphere. The comparison is to jobs in the village that the owner/manager qualifies for if all economic activity is within the village, but to jobs within the nation if the owner/manager participates in national markets as a consumer and labor markets are fluid.
		Education Access	• **5**: Higher education is accessible;<br>• **4**: High school level education or advanced technical training is accessible;<br>• **3**: Middle school level education or simple technical training is accessible;<br>• **2**: Basic literacy and arithmetic training is accessible;<br>• **1**: Formal education is not accessible	Measure is based on the highest level of education that is accessible to (available and affordable) the families (i.e., children) of processing owners and managers. Not based on the actual educational attainment levels of current processing owners/managers.
		Access to Health Care	• **5**: Global standard treatment for illness is accessible;<br>• **4**: Licensed doctors provide trauma, surgical and drug treatments;<br>• **3**: Nurses or medical practitioners provide emergency and routine drug treatments;<br>• **2**: Basic and simple drug treatment is accessible;<br>• **1**: Medical or drug treatment is not accessible	Measure is based on the quality of health care that is accessible to (available and affordable) the processing owners/managers and their families.
		Social Standing of Processing Managers	• **5**: Among the most respected in the community, comparable with civic and religious leaders and professionals, such as doctors and lawyers;<br>• **4**: Comparable to management and white collar jobs;<br>• **3**: Comparable to skilled labor jobs;<br>• **2**: Comparable to unskilled blue collar or service jobs;<br>• **1**: Among the least respected, such as slaves or indentured servants	Measure is based on the social standing of processing owners/managers within the community where they spend the majority of their time.
		Nonresident Ownership of Processing Capacity	• **5**: 95-100% local;<br>• **4**: 70-95% local;<br>• **3**: 35-70% local;<br>• **2**: 5-35% local;<br>• **1**: Virtually no local processing ownership	Proportion of processing owners/managers who are local. "Local" is defined as coming from, and spending their earnings within, the local fishing community. Nationals who are transient nonresidents, or considered outsiders [illegible]
		Earnings Compared to Regional Average Earnings	• **5**: More than 50% above the regional average;<br>• **4**: Between 10 and 50% above regional average;<br>• **3**: Within 10% above the regional average;<br>• **2**: Between 50% and 90% of the regional average;<br>• **1**: Less than half of the regional average	Ratio of annual earnings per processing worker to the regional average earnings. Note that this is earnings from all sources and not just fishing.
		Worker Wages Compared to Non-fishery Wages	• **5**: More than 50% above the regional average;<br>• **4**: Between 10 and 50% above regional average;<br>• **3**: Within 10% above the regional average;<br>• **2**: Between 50% and 90% of the regional average;<br>• **1**: Less than half of the regional average	Ratio of processing worker's average daily wage in this fishery to the average daily wage in the worker's alternate occupations within their economic sphere. The comparison is to jobs in the village that the worker qualifies for if all economic activity is within the village, but to jobs within the nation if the worker participates in national markets as a consumer [illegible]

Metric	IOTC (n=7;N=4)				IATTC (n=4;N=4)				WCPFC (n=14;N=8)								ICCAT (n=4;N=3)			CCSBT (n=2;N=2)		
	DW-PS-SKJ (n=1; A)	SE-PL-SKJ (n=1; A)	SE-GNLL-SKJmsp (n=1; C)	SE-LL/HL-YFTmsp (n=4; B)	I-PS-SKJ (n=1; B)	A-YFT/BETmsp (n=1; B)	I-PLTR-ALB (n=1; A)	I-PS-BFT (n=1; A)	PNA:FSMA-I-PS-SKJ (n=, 3*; A)	I-PS-SKJ (n=1; B)	USTr-DW-PS-SKJ (n=2; A)	DW-PS-SKJ (n=2; B)	DW-LL-ALLmsp (n=2; A)	I-LL-ALLmsp (n=2; A)	SE-LL-YFT (n=1; B)	A-LL-YFT/msp (n=1; B)	DW-PS-SKJ (n=1; A)	DW-LL-ALLmsp (n=2; A)	I-PS-BFT (n=1; A)	DW-LL-ALLmsp (n=1; A)	I-PS-SBF (n=1; A)	PNA:FSMA-SE-PS-SKJ (n=2; A)
	Aggregate Score	Aggregate Score	Aggregate Score	Aggregate Score	Aggregate Score	Aggregate Score	Aggregate Score	Aggregate Score	Aggregate Score	Aggregate Score	Aggregate Score	Aggregate Score	Aggregate Score	Aggregate Score	Aggregate Score	Aggregate Score	Aggregate Score	Aggregate Score	Aggregate Score	Aggregate Score	Aggregate Score	Aggregate Score
Percentage of Stocks Overfished	5.0	5.0	5.0	5.0	3.0	3.0	5.0	1.0	5.0	3.0	3.0	3.7	3.0	3.0	4.0	3.0	4.0	1.0	1.0	1.0	1.0	5.0
Source of Capital	4.0	3.0	2.0	3.6	4.0	4.0	5.0	5.0	3.0	4.0	3.4	3.7	4.3	5.0	5.0	4.0	4.0	4.3	5.0	5.0	5.0	3.0
Age of Facilities	3.0	2.0	3.0	4.3	3.0	2.0	3.0	4.0	4.0	3.0	1.6	2.4	3.0	3.0	3.0	4.0	3.0	3.0	4.0	3.0	4.0	1.7
Earnings Compared to Regional Average Earnings	5.0	5.0	3.0	5.0	5.0	5.0	5.0	5.0	5.0	5.0	5.0	5.0	5.0	5.0	3.0	5.0	5.0	5.0	5.0	5.0	5.0	4.4
Manager Wages Compared to Non-fishery Wages	5.0	5.0	3.0	5.0	5.0	5.0	5.0	5.0	5.0	5.0	5.0	5.0	5.0	5.0	2.0	5.0	5.0	5.0	5.0	5.0	5.0	3.7
Education Access	5.0	4.0	4.0	4.0	5.0	5.0	5.0	5.0	3.0	5.0	5.0	5.0	5.0	5.0	4.0	4.0	5.0	5.0	5.0	5.0	5.0	3.4
Access to Health Care	5.0	3.0	4.0	4.0	4.0	4.0	5.0	4.0	2.0	5.0	4.6	5.0	5.0	5.0	4.0	3.0	5.0	5.0	5.0	5.0	5.0	3.1
Social Standing of Processing Managers	4.0	4.0	2.0	4.0	5.0	5.0	4.0	5.0	5.0	4.0	4.6	5.0	5.0	5.0	4.0	4.0	4.0	5.0	5.0	5.0	5.0	4.0
Nonresident Ownership of Processing Capacity	5.0	5.0	5.0	4.3	4.0	5.0	5.0	5.0	2.0	3.0	3.6	5.0	5.0	5.0	2.0	3.0	1.0	5.0	5.0	5.0	5.0	2.0
Earnings Compared to Regional Average Earnings	3.0	3.0	2.0	3.4	3.0	3.0	3.0	5.0	2.0	3.0	2.4	2.7	3.3	4.0	3.0	3.0	3.0	3.3	3.0	4.0	3.0	4.4
Worker Wages Compared to Non-fishery Wages	2.0	3.0	2.0	3.4	3.0	3.0	3.0	5.0	2.0	4.0	3.0	3.0	3.0	3.0	2.0	5.0	3.0	3.0	3.0	3.0	3.0	2.3

MANAGEMENT SYSTEM

SCALE OF PRODUCTION & GEAR

Indicator	Dimension	Metric	Score System	Additional Explanation
	Processing Workers	Percentage of Stocks Overfished	• **5**: None overfished;<br>• **4**: 1-25% of stocks overfished;<br>• **3**: 26-50% overfished;<br>• **2**: 51-75% overfished;<br>• **1**: 76-100% overfished	Percentage of commercial stocks within the management authority's jurisdiction that are considered to be overfished, to be experiencing overfishing, or whose stock status is generally unknown. (Degree of overfishing and current trend are the next questions.) Single stock fisheries will be scored 1 or 5.
		Education Access	• **5**: Higher education is accessible;<br>• **4**: High school level education or advanced technical training is accessible;<br>• **3**: Middle school level education or simple technical training is accessible;<br>• **2**: Basic literacy and arithmetic training is accessible;<br>• **1**: Formal education is not accessible	Measure is based on the highest level of education that is accessible to (available and affordable) the families (i.e., children) of processing workers. Not based on the actual educational attainment levels of current processing workers.
		Access to Health Care	• **5**: Global standard treatment for illness is accessible;<br>• **4**: Licensed doctors provide trauma, surgical and drug treatments;<br>• **3**: Nurses or medical practitioners provide emergency and routine drug treatments;<br>• **2**: Basic and simple drug treatment is accessible;<br>• **1**: Medical or drug treatment is not accessible	Measure is based on the quality of health care that is accessible to (available and affordable) the processing workers and their families.
		Social Standing of Processing Workers	• **5**: Among the most respected in the community, comparable with civic and religious leaders and professionals, such as doctors and lawyers;<br>• **4**: Comparable to management and white collar jobs;<br>• **3**: Comparable to skilled labor jobs;<br>• **2**: Comparable to unskilled blue collar or service jobs;<br>• **1**: Among the least respected, such as slaves or indentured servants	Measure is based on the social standing of workers within the community where they spend the majority of their time.
		Proportion of Nonresident Employment	• **5**: 95-100% local;<br>• **4**: 70-95% local;<br>• **3**: 35-70% local;<br>• **2**: 5-35% local;<br>• **1**: Virtually no local workers	Proportion of processing workers who are local. "Local" is defined as coming from, and spending their earnings within, the local fishing community. Nationals who are transient nonresidents, or considered outsiders in the fishing community, are not local
		Worker Experience	• **5**: More than 10 years (skilled career workers);<br>• **4**: 5-10 years;<br>• **3**: 3-5 years;<br>• **2**: 1-3 years;<br>• **1**: 0 full years of experience (mostly new workers each season)	Average years of experience of processing workers.

	IOTC (n=7;N=4)				IATTC (n=4;N=4)				WCPFC (n=14;N=8)								ICCAT (n=4;N=3)			CCSBT (n=2;N=2)		
Metric	DW-PS-SKJ (n=1; A)	SE-PL-SKJ (n=1; A)	SE-GNLL-SKJmsp (n=1; C)	SE-LL/HL-YFTmsp (n=4; B)	I-PS-SKJ (n=1; B)	A-YFT/BETmsp (n=1; B)	I-PLTR-ALB (n=1; A)	I-PS-BFT (n=1; A)	PNA:FSMA-I-PS-SKJ (n=, 3*; A)	I-PS-SKJ (n=1; B)	USTr-DW-PS-SKJ (n=2; A)	DW-PS-SKJ (n=2; B)	DW-LL-ALLmsp (n=2; A)	I-LL-ALLmsp (n=2; A)	SE-LL-YFT (n=1; B)	A-LL-YFT/msp (n=1; B)	DW-PS-SKJ (n=1; A)	DW-LL-ALLmsp (n=2; A)	I-PS-BFT (n=1; A)	DW-LL-ALLmsp (n=1; A)	I-PS-SBF (n=1; A)	PNA:FSMA-SE-PS-SKJ (n=2; A)
	Aggregate Score	Aggregate Score	Aggregate Score	Aggregate Score	Aggregate Score	Aggregate Score	Aggregate Score	Aggregate Score	Aggregate Score	Aggregate Score	Aggregate Score	Aggregate Score	Aggregate Score	Aggregate Score	Aggregate Score	Aggregate Score	Aggregate Score	Aggregate Score	Aggregate Score	Aggregate Score	Aggregate Score	Aggregate Score
Percentage of Stocks Overfished	5.0	5.0	5.0	5.0	3.0	3.0	5.0	1.0	5.0	3.0	3.0	3.7	3.0	3.0	4.0	3.0	4.0	1.0	1.0	1.0	1.0	5.0
Education Access	2.0	2.0	2.0	3.0	2.0	3.0	2.0	4.0	1.0	2.0	2.0	2.4	3.3	4.0	3.0	3.0	2.0	3.3	3.0	4.0	2.0	2.0
Access to Health Care	3.0	3.0	2.0	3.4	3.0	3.0	5.0	5.0	2.0	3.0	3.4	3.7	4.3	4.3	4.0	3.0	3.0	4.0	4.0	5.0	4.0	2.7
Social Standing of Processing Workers	4.0	3.0	3.0	3.4	4.0	4.0	5.0	4.0	2.0	4.0	4.0	4.4	5.0	5.0	4.0	2.0	3.0	5.0	5.0	5.0	4.0	3.1
Proportion of Nonresident Employment	3.0	2.0	5.0	3.8	5.0	5.0	3.0	5.0	5.0	5.0	3.0	3.4	4.3	5.0	4.0	5.0	3.0	4.3	3.0	5.0	3.0	4.3
Worker Experience	3.0	3.0	5.0	3.0	4.0	3.0	4.0	4.0	1.0	2.0	2.0	3.1	3.5	5.0	3.0	3.0	2.0	3.7	3.0	5.0	3.0	1.3
